# Dynamic male mouse gut microbiota signature linked to improved wound healing of a novel salecan hydrogel dressing

**DOI:** 10.3389/fbioe.2025.1584976

**Published:** 2025-08-21

**Authors:** Guangping Luo, Tongtong Zhang, Yanan Jiang, Yuan Qin, Pengfei Chen, Junqing Hu

**Affiliations:** ^1^ Obesity and Metabolism Medicine-Engineering Integration Laboratory, Department of General Surgery, The Affiliated Hospital of Southwest Jiaotong University, The Third People’s Hospital of Chengdu, Chengdu, China; ^2^ The Center for Obesity and Metabolic Diseases, Department of General Surgery, The Affiliated Hospital of Southwest Jiaotong University, The Third People’s Hospital of Chengdu, Chengdu, China; ^3^ Medical Research Center, The Affiliated Hospital of Southwest Jiaotong University, The Third People’s Hospital of Chengdu, Chengdu, China; ^4^ School of Food and Bioengineering, Xihua University, Chengdu, China

**Keywords:** wound repair, skin regeneration, faecal microbiota, bacteria, salecan hydrogel

## Abstract

Salecan-based hydrogel (thereafter called Sal-hydrogel) dressings and gut microbiota have been associated with enhanced wound healing. However, the relationship between these two factors remains unclear. This study investigated the dynamic characteristics of the intestinal microbiota in relation to the Sal-hydrogel dressings and their role in promoting wound healing. Fecal samples were collected at day 0, 3, 7, and 12 after wounds were inflicted on 48 mice, which were treated with either Sal-hydrogel, *Staphylococcus aureus*, or a combination of *S. aureus* and Sal-hydrogel, using a full-thickness skin perforation wound model. The samples were subjected to 16S rRNA V3-V4 gene sequencing. The results indicated a general trend for Shannon diversity of the intestinal microbiota to increase by day 3 following injury. On the final day, the diversity in both the hydrogel group and the *S. aureus* plus hydrogel group was significantly higher compared to the control group (*p* < 0.05). Additionally, the bacterial community structure in the mouse gut exhibited substantial changes when comparing the hydrogel-treated groups to the controls (hydrogel group: *p* = 0.027; *S. aureus* plus hydrogel group: *p* = 0.039). The genus *uncultured_*Oscillospiraceae, which was significantly associated with wound closure (R-squared = 0.2154, *p* = 6.657e-05), was found to be dominant in the gut of the hydrogel group during the wound healing process. Overall, our findings suggest that significant and rapid alterations in gut microbiota occur in response to skin injury and wound infection. The enhanced wound healing properties of the Sal-hydrogel dressing are associated with increased intestinal microbiota diversity and the presence of the bacterium *uncultured_*Oscillospiraceae.

## Introduction

Nonhealing skin wounds represent a significant global healthcare challenge. Under normal conditions, skin damage triggers precisely regulated inflammatory and tissue-regeneration responses that facilitate the elimination of pathogens and the maintenance of barrier function (Proksch et al., 2008; Kabashima et al., 2019; Bernatchez and Bichel, 2023). However, in the case of bacterial infections, these responses may malfunction, either failing to activate or functioning abnormally, which can lead to complications in tissue formation and lasting damage to the epidermal barrier (Kabashima et al., 2019). To address this issue, wound dressings are typically applied to protect the wound from further trauma and invasion by pathogenic microorganisms, thereby providing optimal conditions for wound healing. Currently, several types of wound dressings are available, including hydrogels used in moist wound therapies (Liang et al., 2021; Nuutila and Eriksson, 2021). Salecan, a microbial polysaccharide produced by *Agrobacterium* sp. ZX09, is a novel water-soluble extracellular glucan that is particularly appealing for biomaterial applications due to its biodegradability, low cost, and other advantageous properties (Qi et al., 2019). Our previous study demonstrated that salecan-based hydrogel (Sal-hydrogel) dressings effectively promote wound healing and tissue regeneration (Deng et al., 2024). Therefore, it is anticipated that Sal-hydrogel will be utilized as a wound dressing. However, it exhibited suboptimal mechanical properties as a single-component system. Consequently, in this study, we engineered a composite hydrogel (Sal/DA/AgNPs/PVA).

The human microbiota is essential for overall health and significantly influences various bodily functions, including wound healing. When the balance of the microbiota is disrupted, pathogenic microbes are more likely to infect wounds, leading to increased inflammation and impaired healing. Studies have shown that the healing abilities of germ-free mice and mice treated with antibiotics are compromised, providing further evidence that the skin microbiota plays a critical role in wound healing (Di Domizio et al., 2020; Wang et al., 2021). In addition to skin microbiota, gut microbiota also plays an important role in wound healing (Bu et al., 2023; Wade et al., 2023; Zheng et al., 2023). The important role of gut microbiota in disease and health has also been widely reported (Fan and Pedersen, 2021; Wu et al., 2021), and it is generally believed that gut microbiota is closely related to physiological functions such as host metabolism, inflammation, and immunity (Kamada et al., 2013; Zheng et al., 2020; Schröder, 2022). Therefore, targeting the microbiota to promote wound healing through hydrogel dressings is a promising approach. And it is expected that the combination of intestinal microbiota and Sal-hydrogel will be applied to the field of wound healing medicine.

In this context, the current study aims to investigate the characteristics of the intestinal microbiota associated with the novel Sal-hydrogel dressing in promoting wound healing and preventing infection. Additionally, the study will analyze the changes in the intestinal microbiota throughout the healing process and examine the characteristics of the intestinal microbiota at the conclusion of healing. Furthermore, the research will explore potential microbial markers that could enhance wound healing and analyze the interactions by which the intestinal microbiota’s response to Sal-hydrogel treatment impacts healing in mice, and potentially in patients with chronic non-communicable diseases, such as diabetic foot ulcers, in the future. Ultimately, the data presented here will be valuable for the development of microbiome-based Sal-hydrogel products for wound treatment.

## Materials and methods

### Animals, wound model, and treatments

Male C57/BL6 mice (six to eight weeks old) were purchased from Dossy Experimental Animals Ltd. (Chengdu, China). Forty-eight mice were equally divided into four groups, with similar average body weights of 12 mice each. Before the treatments and wound assessments, the mice were randomly grouped. The mice were housed in animal care facilities that were free of specific pathogens (i.e., bacteria, viruses, or other disease-causing microorganisms). The mice had free access to drinking water and normal chow diet. To establish the wound model, wounds of 10 mm diameter were made on the backs of mice with the use of a punch under general anesthesia. A Matrx VIP 3000 Vaporizer (Midmark, Miamisburg, OH, United States) was utilized to generate isoflurane vapor for the inhalational anesthesia of mice, initially at a concentration of 3% and subsequently at 1%–3% during the procedure with 0.5 L/min O_2_. The depth of anesthesia was monitored by breathing rate. On the first day (Day 0), the groups received the following treatments: (1) control: mice in the control group only had wound models; (2) hydrogel: mice were coated with Sal-hydrogel dressings over the wounds; (3) chronic infection model (*S. aureus*): *S. aureus* (subsp. *aureus* strain Wichita, ATCC 29213, 1 × 10^6^ CFU/mL, 200 μL) were placed over the wounds (Klopfenstein et al., 2021); (4) *Staphylococcus aureus* + hydrogel: mice wounds were treated with both *S. aureus* (1 × 10^6^ CFU/mL, 200 μL) and Sal-hydrogel dressings.

Sal-hydrogel dressings used in current study are semi-occlusive and composed of complex hydrophyllic polymers (Sal/DA/AgNPs/PVA) with a high (>90%) water content. This composite hydrogel was developed by incorporating the linear water-soluble polymer polyvinyl alcohol (PVA), which is known for its excellent biocompatibility and crystallinity, to enhance the mechanical properties of the Salacan hydrogel. Additionally, we introduced polydopamine (PDA) for its adhesive characteristics. Furthermore, by leveraging the reducing capability of PDA, silver ions (Ag^+^) were *in situ* reduced to silver nanoparticles within the gel matrix, thereby augmenting the antibacterial properties of the composite hydrogel. The inhibition rate of the Sal-hydrogel against *S. aureus* was 99.5% *in vitro* (Supplementary Figure S1).

**FIGURE 1:**
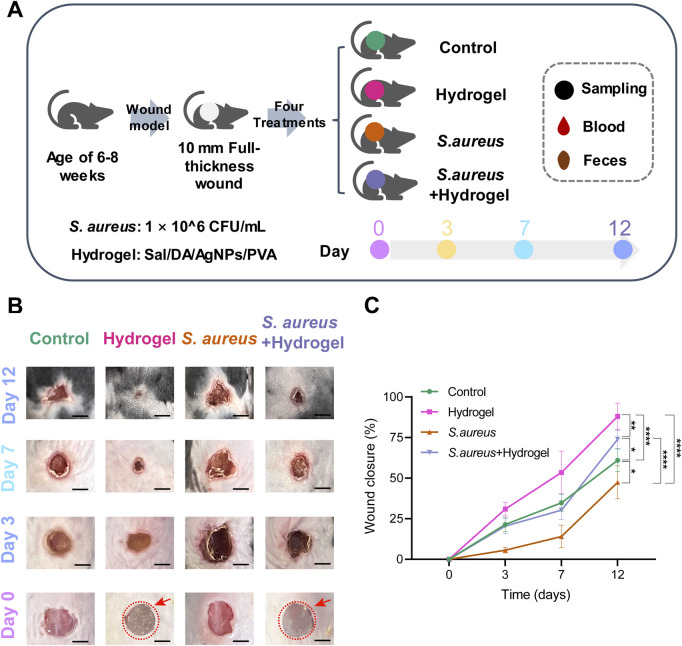
Overview of the design of the current study. **(A)** Diagram of study design. **(B)** The representative images illustrated the effect of Sal-hydrogel dressings on skin wound healing at day 3, 7, and 12. Day 0 demonstrated the creation of the wound and the application of external Sal-hydrogel dressings. The salecan hydrogels applied to the wound were indicated with a red circle and arrow in both the hydrogel and *Staphylococcus aureus* and hydrogel groups. Scale bar, 0.5 cm. **(C)** A comparison of wound closure among the four groups at different time points was conducted using a two-way ANOVA followed by a *post hoc* Tukey HSD test (n = 4 per group/time). *p < 0.05, **p < 0.01, ****p < 0.0001. Sal: Salecan; DA: Dopamine hydrochloride; AgNPs: Silver nanoparticles; PVA: Polyvinyl alcohol.

Each wound received an application of 0.2 mL of Sal-hydrogel, approximately 2 mm thick, ensuring full coverage in groups (2) and (4). After treatment, mice were caged by groups. To avoid excessive wound disturbance, minimize biofilm reformation, and balance infection control with healing, the dressings were changed every 3 days (Schierle et al., 2009; WHO, 2018; Senneville et al., 2024). Fecal samples were collected (in total 64 samples, each timepoint 4 mice/group) from mice at day 0, 3, 7, and 12 (Figure 1A). During the modeling on Day 0, to ensure consistency among the model mice, wounds were monitored by measuring the diameters. On Days 3, 7, and 12, the wounds were photographed, standardized using a scale bar in the images, and the wound areas (mm^2^) were calculated using ImageJ software (version 1.54). The healing rate was calculated using the following formula:

$$\frac{Initial\;wound\;area-Wound\;area\;at\;the\;observation\;time\;point}{Initial\;wound\;area}\times 100\%$$

### Ethics approval

All animal experiments using C57/BL6 mice were conducted in accordance with the Xihua University’s Regulations for Animal Care and Use, approved by the Animal Experiment Ethics Committee, and we confirmed the study was reported in accordance with ARRIVE guidelines, the mice were placed in a darkened home cage and euthanized using carbon dioxide (CO^2^) with a displacement rate of 50% of the cage volume per minute.

### Microbial DNA extraction, PCR amplification, and sequencing

For sequencing, genomic DNA from the fecal samples was extracted by Soil DNA Kit according to the manufacturer’s instructions (Omega Bio-Tek, GA, United States). The quality of the genomic DNA was tested using 1% agarose gel electrophoresis, and the concentration and purity of the DNA were determined using the NanoDrop 2000. Once checked, the V3-V4 region of the bacterial 16S rRNA was amplified using the primers 338F (ACT​CCT​ACG​GGA​GGC​AGC​AG), 806R (GGACTACNNGGGTATCTAAT), and the polymerase chain reaction was performed in a 20 μL reaction system. The amplification products were purified using AMPure^®^ PB beads (Pacific Biosciences, CA, United States) and quantified using the QuantiFluor-ST (Promega, WI, United States). The PE libraries were constructed using the NEXTFLEX Rapid DNA-Seq Kit (Bioo Scientific, TX, United States) and were sequenced using the Illumina Miseq PE300 platform.

### Sequencing data analysis

The sequencing data of bacterial 16S rRNA gene sequence were imported into QIIME 2 (v2022.2) for preprocessing, denoising, diversity analyses and taxonomy classification (Bolyen et al., 2019). The raw sequence data were demultiplexed and quality filtered using the q2-demux plugin. Then, denoising was done with DADA2 (Callahan et al., 2016) using the q2-dada2 plugin. Amplicon sequence variants (ASVs) with a frequency of one were filtered. All the remaining ASVs were aligned to mafft (Katoh et al., 2002) using the q2-alignment plugin, and used to construct a phylogeny with fasttree2 (Price et al., 2010) using the q2-phylogeny plugin. The Shannon’s entropy diversity metric (Shannon, 1997), the weighted UniFrac (Lozupone et al., 2007) and Principal Coordinate Analysis (PCoA) were calculated using q2-diversity after samples were rarefied (without replacement) to 3,450 sequences per sample. To prevent the loss of samples due to rarefaction at a higher value, we selected 3,450 as the rarefaction depth. Alpha rarefaction plotting indicated that our chosen rarefaction depth did not significantly affect diversity (Supplementary Figure S2). Taxonomy of the ASVs were assigned using the q2-feature-classifier (Bokulich et al., 2018) classify-sklearn naïve Bayes taxonomy classifier against the Silva 138 99% OTUs reference sequences (Kaehler et al., 2019).

### SparCC network construction and analysis

The iNAP (Integrated Network Analysis Pipeline) was employed to generate a global SparCC network of bacterial interactions at the genus level (Feng et al., 2022). Firstly, the majority step was to identify and filter genera that were less frequent than expected by chance in at least half of the samples. Secondly, SparCC correlations (Friedman and Alm, 2012) were calculated and pseudo p-values were estimated through a bootstrap approach. Finally, the bipartite network matrix and visualization output from SparCC results were generated. The correlated genus pairs were selected when the absolute value of the sparse correlation was |r| >0.4 and *p* < 0.05 (two sided). Visualization and analysis of the network were conducted using Gephi (v0.9.2) (Bastian et al., 2009).

### Prediction of metagenome pathways

To predict the functions of the bacterial metagenome by 16S rRNA gene data, the PICRUSt2 (v2.5.0) workflow was applied (Douglas et al., 2020). The entire PICRUSt2 pipeline was run using a single script, picrust2_pipeline.py. This script performed each of the four critical steps of PICRUSt2: (1) placement of reads, (2) genome prediction for hidden states, (3) metagenome prediction, (4) pathway-level predictions. NSTI (nearest-sequenced taxon index) was calculated for each input ASV by default and excluded any with an NSTI >2.

### Statistical analysis

Differences in alpha and beta diversity within each group using the non-parametric Kruskal Wallis and Permutational multivariate analysis of variance (PERMANOVA), with 999 permutations for the PERMANOVA test. Differentially abundant taxa were identified by linear discriminant analysis (LDA) effect size (LEfSe) with corrections for independent comparisons. A chi-square (χ2) test was used to determine if there was a difference in the number of correlations between the groups. A two-way ANOVA with *post hoc* Tukey HSD Test was applied to compare the difference in the wound closure and taxonomic composition of the groups. F-statistic was applied to test the linear association between taxonomic abundance and wound closure. Kruskal Wallis test with Benjamini–Hochberg (BH) adjustment was also used to compare the difference in the predicted pathways between two groups.

## Results

### The salecan-hydrogel dressings effectively improve wound healing

The characteristics and differences in wound healing among the four groups were initially identified (Figures 1B,C). By day 12, the wounds in the hydrogel group (77.52%–96.66%, mean 88.07%) had nearly healed completely, while those in the control group remained unhealed (54.98%–69.17%, mean 60.97%). Similarly, the wounds in the *S. aureus* and hydrogel group (65.81%–78.63%, mean 73.93%) were almost fully healed, whereas the wounds in the *S. aureus* group (35.38%–57.22%, mean 47.42%) remained unhealed (*p* < 0.0001). Notably, the wounds in the *S. aureus* group exhibited poorer healing compared to the other three groups. However, the wounds in the hydrogel group healed more effectively than those in the control group (*p* < 0.0001), but not as well the *S. aureus* and hydrogel group (*p* = 0.0207). These results indicate that, compared to the control group, the wound healing rate of the mice in the hydrogel treatment groups of mice was significantly enhanced.

### Better wound healing is associated with higher gut microbial diversity

**FIGURE 2:**
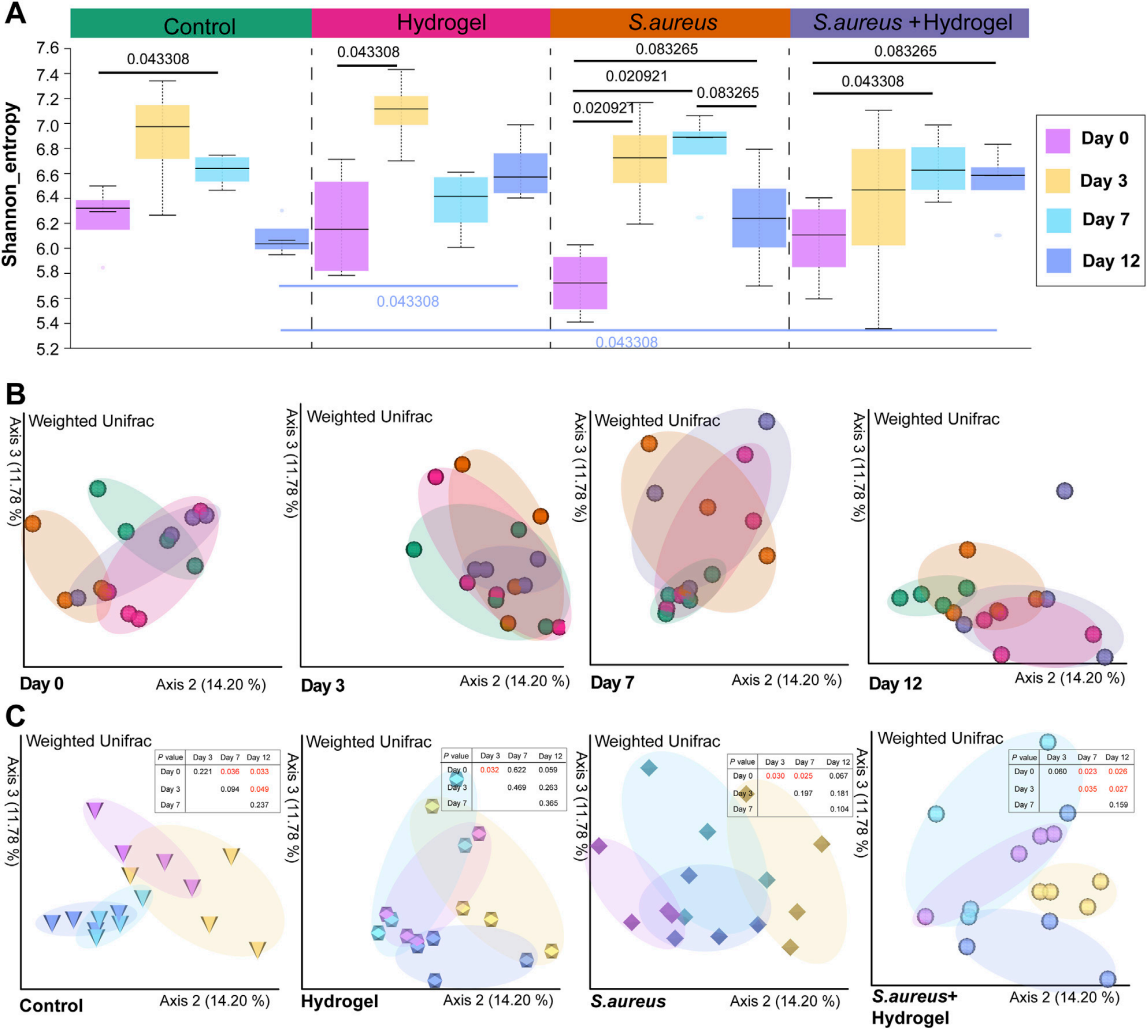
Alteration of gut microbial diversity during skin wound healing. **(A)** Gut microbial Shannon diversity among control, hydrogel, *Staphylococcus aureus* and *Staphylococcus aureus* and hydrogel groups at day 0, 3, 7, and 12 (n = 4 per group/time). The statistical analysis was performed using the Kruskal Wallis test. **(B)** Principal coordinate analysis (PCoA) of the weighted Unifrac distance among four groups at different time points (n = 4 per group/time). **(C)** Principal coordinate analysis (PCoA) of the weighted Unifrac distance among four time points in different groups (n = 4 per group/time). The statistical analysis was performed using a pairwise PERMANOVA test.

The differences in gut microbiota diversity among the four groups were assessed using the 16S rRNA method. On the 12th day, the Shannon diversity index of the hydrogel group (*p* = 0.043) and the *S. aureus* and hydrogel group (*p* = 0.043) was significantly higher than that of the control group (Figure 2A). These results were positively connected with the wound healing rate in the hydrogel treatment group, suggesting that enhanced wound healing properties may be attributed to increased gut microbial diversity in mice.

### Altered microbial diversity and community structure relate to time of wound recovery

Generally, the Shannon diversity of gut microbial communities exhibited an upward trend in all four groups on day 3 following the infliction of wounds (Figure 2A). When compared to day zero, statistically significant differences were observed on either day 3 or day 7 of the treatments (control group: day 7, *p* = 0.043; hydrogel group: day 3, *p* = 0.043; *S. aureus* group: days 3 and 7, *p* = 0.021; *S. aureus* and hydrogel group: day 7, *p* = 0.043). These results were consistent with the changes in microbial community structure, as illustrated in Figure 2C. Importantly, PCoA of the weighted Unifrac distances indicated that, compared to the control group, the bacterial community structures in the gut of mice treated with the hydrogel (hydrogel group: *p* = 0.027; *S. aureus* and hydrogel group: *p* = 0.039) were significantly altered on day 12 after the wounds were inflicted (Figure 2B; Table 1).

**TABLE 1:** PERMANOVA (pseudo-F, permutations 999) test for microbial community structure.

Group	Time	Hydrogel	*S. aureus*	*S. aureus* + hydrogel
Control	Day 0	0.076	0.028	0.034
	Day 3	0.975	0.771	0.078
	Day 7	0.941	0.035	0.141
	Day 12	0.027	0.057	0.039
Hydrogel	Day 0		0.039	0.251
	Day 3		0.610	0.038
	Day 7		0.724	0.740
	Day 12		0.023	0.431
*S. aureus*	Day 0			0.034
	Day 3			0.147
	Day 7			0.879
	Day 12			0.134

### Wound healing may link to longitudinal changes of gut microbiota composition

**FIGURE 3:**
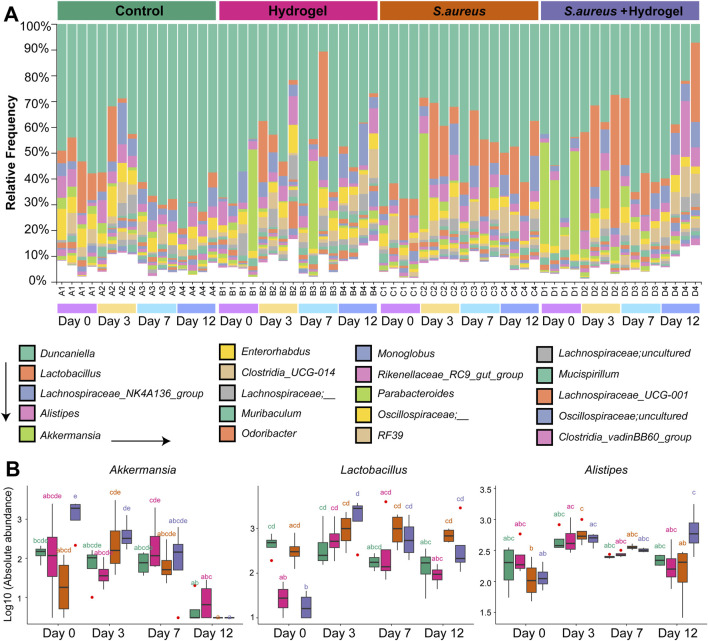
Alteration of gut microbial composition during skin wound healing. **(A)** Bar plot depicted the bacterial relative abundance at the genus level. Top 20 genera were shown. **(B)** Boxplot presented the abundance of genera *Akkermansia*, *Lactobacillus*, and *Alistipes* among four groups at different time points (n = 4 per group/time). Statistic was performed by a two-way ANOVA with *post hoc* Tukey HSD test. Different lowercase letters indicated significant differences (*p* < 0.05).

We next sought to investigate the longitudinal compositional differences in gut microbiota among the four groups. At the phylum level, Bacteroidota, Firmicutes, Verrucomicrobiota, and Actinobacteriota were dominant in the gut of mice in this study, considering both group and time (Supplementary Figure S3). At the genus level, *Duncaniella*, *Lactobacillus*, Lachnospiraceae *NK4A136 group*, *Alistipes*, and *Akkermansia* were the five most prevalent genera in the gut of mice in the current study (Figure 3A). Among these genera (Figure 3B), the abundance of bacteria *Akkermansia* and *Lactobacillus* was significantly higher in the *S. aureus* and hydrogel group compared to the other three groups on day 3 after the wounds were inflicted. *Akkermansia* was particularly abundant in the hydrogel group on day 7. The bacterium *Alistipes* was notably abundant in the *S. aureus* and hydrogel group on day 12.

**FIGURE 4:**
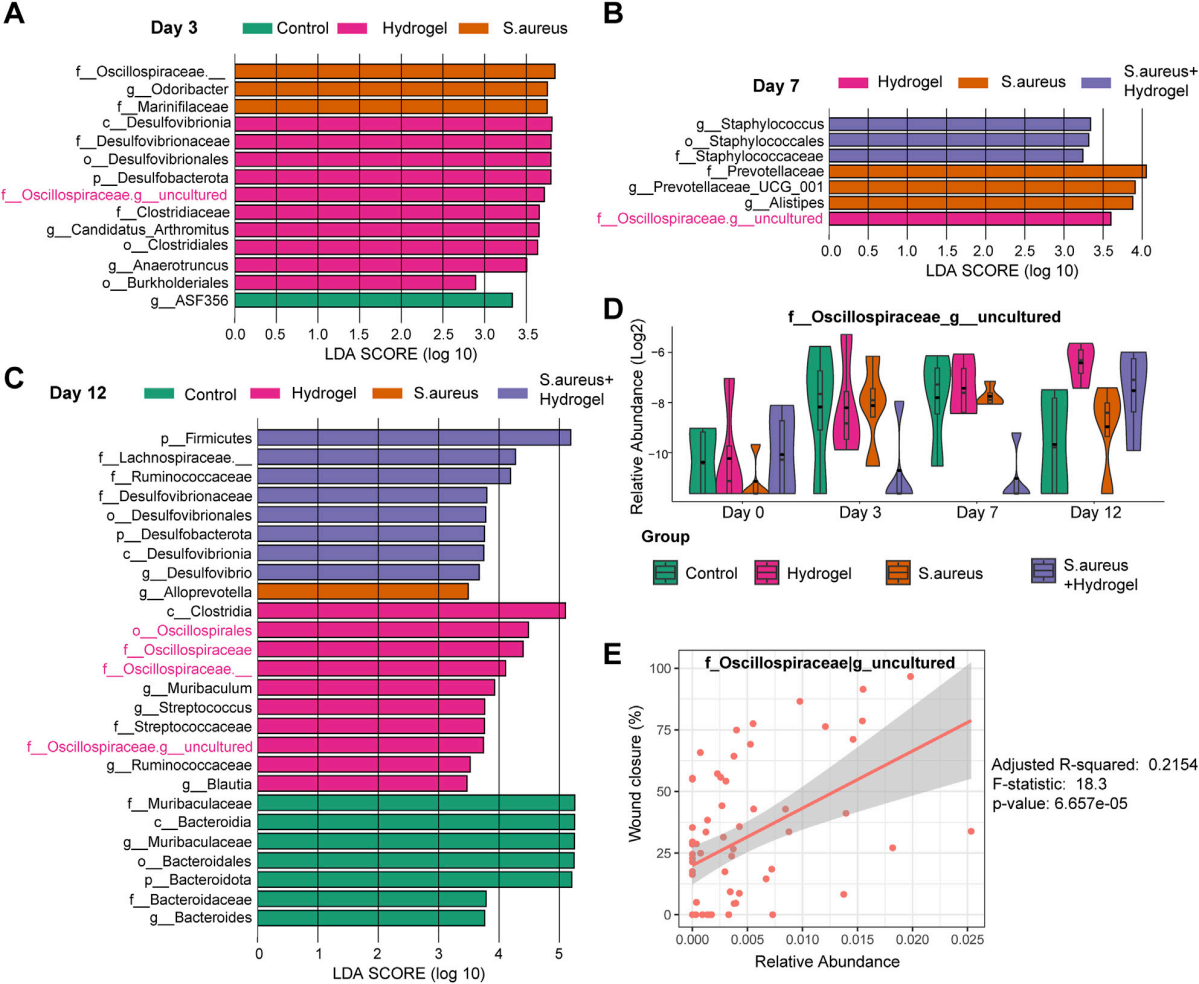
LEfSe analysis for excavating biomarkers to wound healing by hydrogel dressing. **(A–C)** LEfSe barplot showed the different abundance of bacteria among four groups at day 3 **(A)**, 7 **(B)**, and 12 **(C)**. Linear discriminant analysis (LDA) scores were used to identify significant biomarkers that differentiated between groups based on their abundance. The LDA score cutoff (Log10) was set to 2.0. **(D)** Boxplot presented the abundance of genus *uncultured_*Oscillospiraceae among four groups at different time points (n = 4 per group/time). Based on LEfSe results, *uncultured_*Oscillospiraceae was significantly enriched in the mice’s gut of the hydrogel group at all three time points. There was no significant difference among the groups at day 0 (two-way ANOVA with *post hoc* Tukey HSD test). **(E)** Linear association analysis between genus *uncultured_*Oscillospiraceae and wound closure. F statistic was applied. Adjusted R-squared = 0.2154, F-statistic = 18.3, *p*-value = 6.657e-05.

To further excavate the bacterial biomarkers that may contribute to wound healing by hydrogel dressing, LEfSe was performed. On the third day (Figure 4A), Desulfovibrionaceae, *Desulfobacterota*, *Desulfovibrionia*, Desulfovibrionales, *uncultured_*Oscillospiraceae, *Candidatus Arthromitus*, Clostridiales, Clostridiaceae, *Anaerotruncus*, and Burkholderiales were bacterial biomarkers in the hydrogel group. No taxa were identified in the *S. aureus* and hydrogel group. On the seventh day (Figure 4B), only one bacterium *uncultured_*Oscillospiraceae was identified as biomarker in the hydrogel group. No taxa were identified in the control group. On the 12th day (Figure 4C), *Clostridia*, *Oscillospirales*, Oscillospiraceae, Oscillospiraceae*_*, *Muribaculum*, *uncultured_*Oscillospiraceae, *Streptococcus*, Streptococcaceae, and *Blautia* were biomarkers in the hydrogel group. Of note, the genus *uncultured_*Oscillospiraceae was significantly enriched in the mice’s gut of the hydrogel group at all four time points (Figure 4D). Furthermore, the genus *uncultured_*Oscillospiraceae was significantly associated with wound closure (Figure 4E, Adjusted *R*-squared = 0.2154, *p* = 6.657e-05).

### Microbial interactions change significantly among groups during wound healing

To explore the microbial keystone taxa in the gut related to wound healing, we constructed microbial co-occurrence networks. Considering the microbial community structures were similar on day 7 and 12 after the wounds were inflicted (Figure 2C), the data of that two time points was merged as wound healing end time point for network analysis. Meanwhile, the data of the first day of control and hydrogel groups were also merged as control for uninfected group, *S. aureus* and *S. aureus* and hydrogel groups as control for infected group.

**FIGURE 5:**
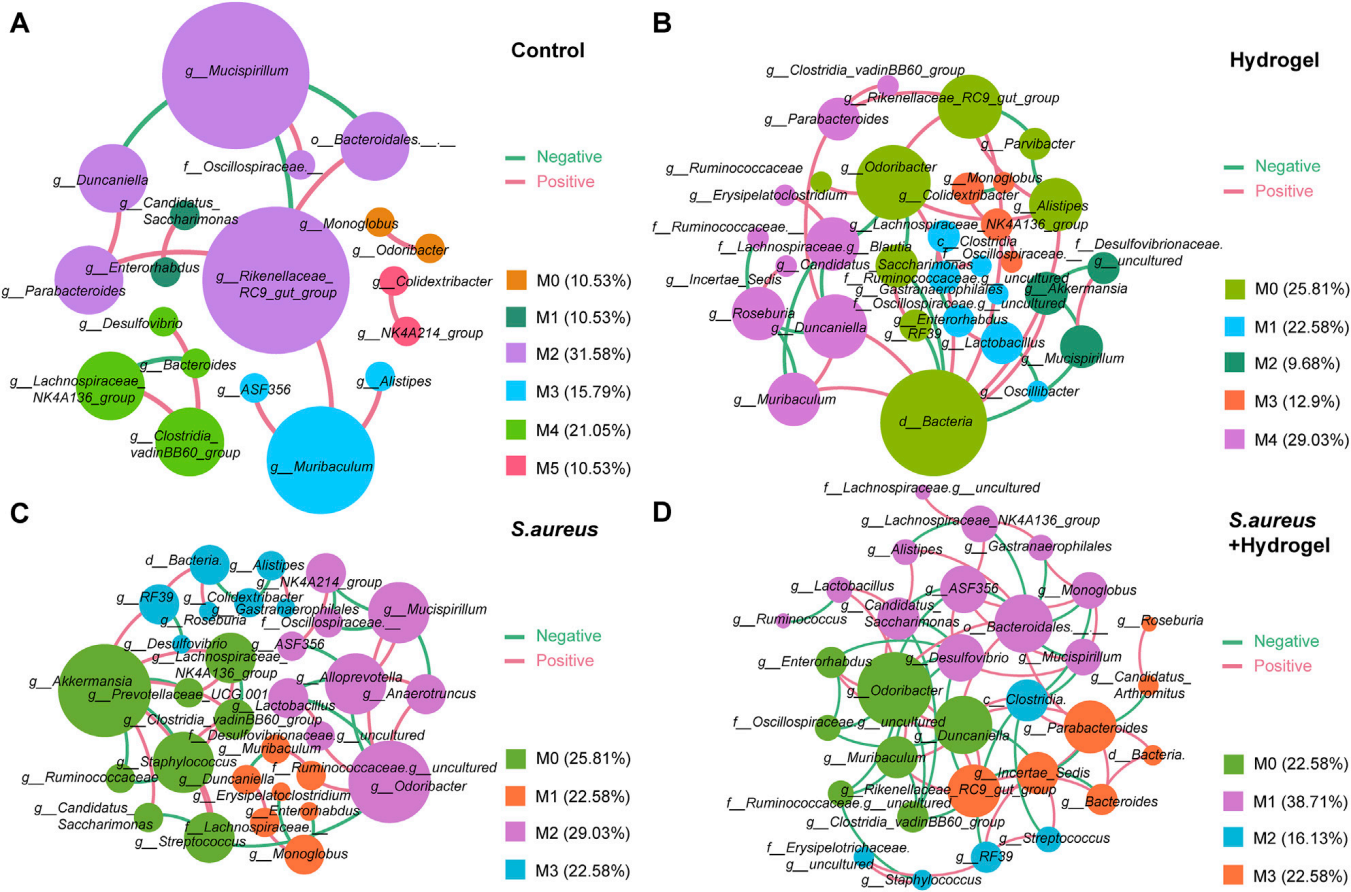
Co-occurrence networks of the bacterial-bacterial correlations in gut. **(A)** Control group. **(B)** Hydrogel group. **(C)** *Staphylococcus aureus* group. **(D)** *Staphylococcus aureus* plus hydrogel group. Each node in the constructed network represented one microbial genus while an edge between two nodes represented a significant association between these microbial taxa (|r| >0.4 and *p* < 0.05). Node size was presented by its degree. Edge colors presented interaction between two nodes. Red edges represented positive correlation, and green represented negative correlation. “M” represented the module of the network.

On Day 0, we identified six modules (M0-5, densely linked communities) and four key communities (keystone taxa: Lachnospiraceae *NK4A136 group*, *Candidatus Arthromitus*, *unclassified* Oscillospiraceae, and *RF39*) in the gut of the uninfected group (Supplementary Figure S4A). In the gut of the infected group, we observed four modules (M0-3) that included two independent communities (keystone taxa: *Streptococcus*, *Lactobacillus*, *RF39*, and *Alistipes*) (Supplementary Figure S4B). By the end of the study, we found six modules (M0-5) and three key communities (keystone taxa: Rikenellaceae RC9 gut group, Mucispirillum, and Muribaculum) in the gut of the control group (Figure 5A). In the hydrogel group, there were five modules (M0-4) and five key communities, including unclassified Bacteria, *Duncaniella*, *Lactobacillus*, and *Akkermansia* (Figure 5B). In the *S. aureus* group, we identified four modules (M0-3) and four key communities (keystone taxa: *Akkermansia*, *Odoribacter*, *Monoglobus*, and *RF39*) (Figure 5C). The *S. aureus* and hydrogel group also exhibited four modules (M0-3) and four key communities, including Bacteroidales, *Odoribacter*, *Parabacteroides*, and *Clostridia* (Figure 5D). Although the control group contained more modules than the hydrogel group, the network in the hydrogel group exhibited a greater number of correlations compared to the control group (41 vs 16; χ2 test, *p* = 8.52E-12; Figures 5A,B and Table 2; Supplementary Tables S1, S2). Additionally, the network in the *S. aureus* and hydrogel group had more correlations than that in the *S. aureus* group (72 vs 44; χ2 test, *p* = 0.00097; Figures 5C,D and Table 2; Supplementary Tables S3, S4).

**TABLE 2:** Summary of bacterial-bacterial co-occurrence networks.

Group	#Node	#Edge	Positive (%)	Negative (%)	#Modularity	*Avg*. Degree	Vs Control	Vs Hydrogel	Vs *S. aureus*
Control	19	16	75	25	6	1.684			
Hydrogel	31	41	56.1	43.9	5	2.645	8.52E-12		
*S. aureus*	31	44	52.27	47.73	4	2.839	5.40E-14	0.65	
*S. aureus* + Hydrogel	31	72	54.17	45.83	4	4.654	3.46E-46	0.00026	0.00097

Network	#Node	#Edge	Positive (%)	Negative (%)	#Modularity	*Avg*. Degree	Vs Control	Vs Hydrogel	
Day 0 No infection	25	31	54.84	45.16	6	2.48	0.0032	0.031	

Network	#Node	#Edge	Positive (%)	Negative (%)	#Modularity	*Avg*. Degree	Vs *S. aureus*	Vs *S. aureus* + Hydrogel	
Day 0 Infected	20	20	50	50	4	2	3.56E-09	1.42E-32	

To further understand the network complexity and keystone taxa involved in wound healing, we compared the co-occurrence networks between the initial and final time points. At the final time point, the network in the hydrogel group exhibited more correlations than that in the corresponding control group (41 vs 31; χ2 test, *p* = 0.031; Figure 5B; Supplementary Figure S4A; Table 2; Supplementary Tables S2, S5). The keystone taxa identified at the final time point included unclassified Bacteria, *Duncaniella*, *Lactobacillus*, and *Akkermansia*, while those at the initial time point were Lachnospiraceae *NK4A136 group*, *Candidatus Arthromitus*, *Akkermansia*, and Ruminococcaceae (Figure 5B; Supplementary Figure S4A). Additionally, the network in the *S. aureus* and hydrogel group demonstrated more correlations than that in the corresponding control group (72 vs 20; χ2 test, *p* = 1.42E-32; Figure 5D; Supplementary Figure S4B; Table 2; Supplementary Tables S4, S6). The keystone taxa at the final time point included Bacteroidales, *Odoribacter*, *Parabacteroides*, and *Clostridia*, whereas those at the initial time point were *RF39*, *Streptococcus*, *Lactobacillus*, and *Alistipes* (Figure 5D; Supplementary Figure S4B). Collectively, these results suggest that enhancing interactions among intestinal bacteria may facilitate improved restoration of a wounded site following hydrogel application.

### Predicted microbial pathways alter markedly among groups during wound healing

**FIGURE 6:**
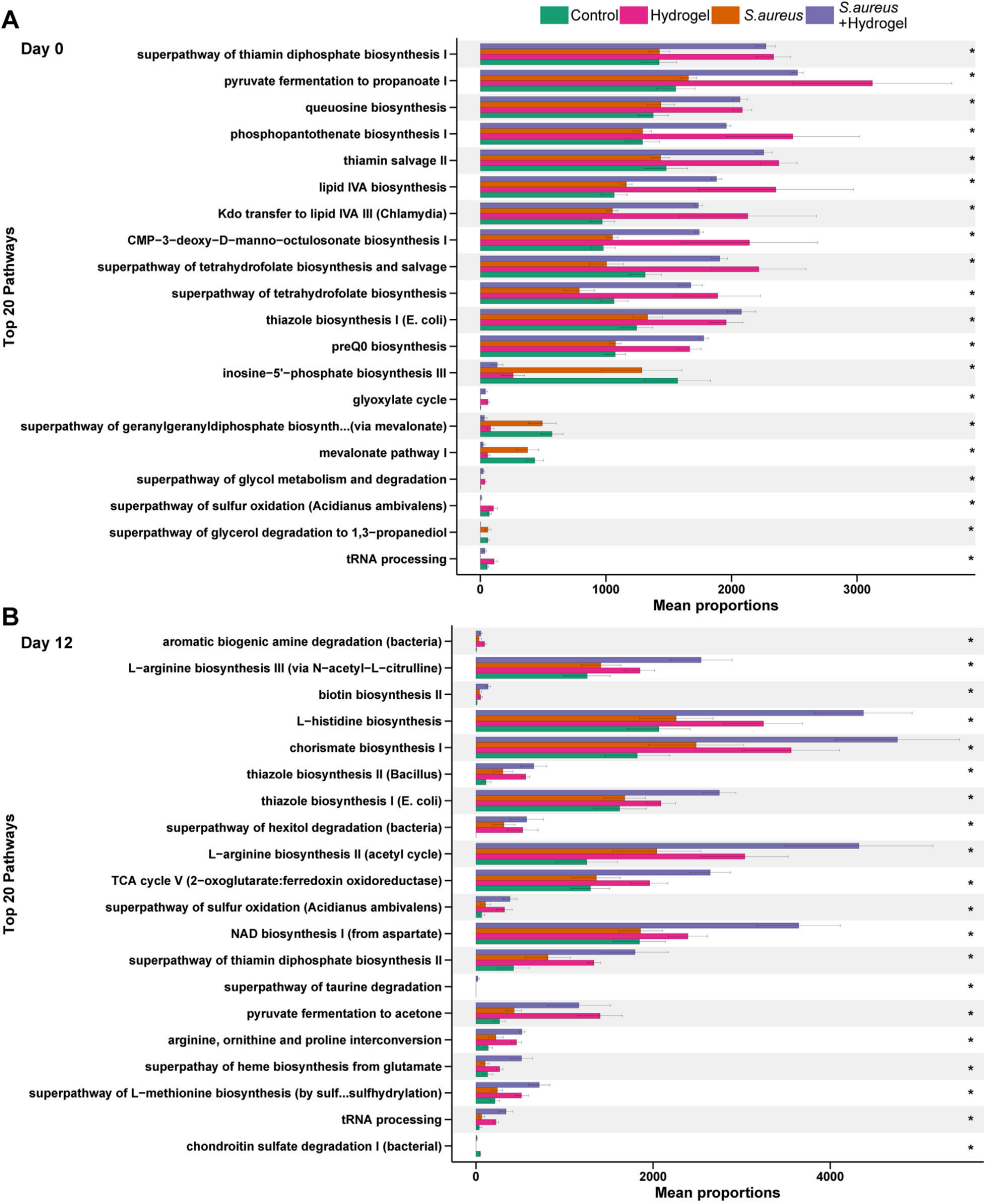
Changes of predicted potential functions of microbiota in gut. The top 20 significant KEGG pathways at day 0 **(A)** and day 12 **(B)** are shown (n = 4 per group/time). Statistic was performed by a Kruskal Wallis test with Benjamini–Hochberg (BH) adjustment. *p adjusted < 0.05. KEGG is developed by Kanehisa Laboratories.

To understand the changes of potential functions among the four groups during wound healing, we further compared the predicted outcomes of bacteria. On the first day (Day 0), 92 pathways in total were significantly changed among the four groups (Supplementary Table S7). As shown in Figure 6A, most of top 20 pathways, including superpathway of thiamin diphosphate biosynthesis I, pyruvate fermentation to propanoate I, and phosphopantothenate biosynthesis I, had the highest proportion in the hydrogel group, following by the *S. aureus* and hydrogel group. On the final day (Day 12), there were one hundred pathways were significantly altered among the four groups (Supplementary Table S8). Although the top 20 pathways were still high proportion in the hydrogel treatment groups, they were higher in the *S. aureus* and hydrogel group than that in the hydrogel group (Figure 6B). L−arginine biosynthesis III (via N−acetyl−L−citrulline), biotin biosynthesis II, L−histidine biosynthesis, chorismate biosynthesis I, L−arginine biosynthesis II (acetyl cycle), and NAD biosynthesis I (from aspartate) were much higher proportion in the *S. aureus* and hydrogel group. The findings suggest the possibility of improving gut bacterial metabolism and thereby accelerate wound repair after hydrogel application.

## Discussion

The skin is the largest organ of the human body, and one of its primary functions is to protect water-rich internal organs from the dry external environment. Maintaining skin integrity and possessing a robust wound healing capacity are essential prerequisites for healthy survival. Furthermore, achieving favorable clinical outcomes in wound management can present significant challenges and burdens to healthcare systems. Surgical injuries rank as the leading source of related expenditures, followed by diabetes-related foot ulceration (Almadani et al., 2021). Therefore, promoting wound healing and reducing the likelihood of lesions are critically important for clinical treatment and patient health. Our previous and preliminary studies have found that Sal-hydrogel dressings can effectively promote wound healing and tissue regeneration in rat and mice, indicating that Sal-hydrogel has great application prospects in skin repair, wound healing, and other medical fields (Deng et al., 2024). The current study determines the intestinal microbiota changes associated with the novel Sal-hydrogel dressing in wound healing and antibacterial effects. The temporal changes in intestinal microbiota and the characteristics of intestinal microbiota at the end of the healing process were analyzed, with the full-thickness skin perforation model used to create a wound. Our study identifies potential intestinal landmark bacteria that can promote wound healing, and illustrates communities interacting during wound repair as well.

High microbial diversity is generally related to human health while a decreased diversity is often related to poor clinical outcomes (Malard et al., 2018). Our study now revises the current evidence of microbial diversity in wound healing, by showing that the higher gut microbiota links to better wound healing rate. And, the expand of gut microbiota diversity occurred on the third day after the wounds were inflicted, which suggests that the first 3 days are critical to wound healing by regulating microbiota diversity. What’s more, our research indicates that Sal-hydrogel dressing was able to boost intestinal microbiota diversity despite exposure of wound to *S. aureus*. *S. aureus* is recently demonstrated to drive itch and scratch-induced skin damage through a V8 protease-PAR1 axis (Deng et al., 2023).

Temporal changes in gut microbiota including diversity, community structure, composition and interactions were observed regarding hydrogel treatment or *S. aureus* exposure over wound in our study. At the endpoint (Day 12), the microbial community structure tends to revert to its initial state (Day 0) in both the hydrogel group and the *S. aureus* group as shown in Figure 2C. However, our study shows the community structures are very similar between day 7 and day 12 regarding treatments after the wounds were inflicted. One possible explanation is that the gut microbiota changes very quickly (approximately 3 days) and maintains relative stable in a very short time (approximately 4 days) in current study. In return, this phenomenon indicates that the gut microbiota has very strong ability to resist external perturbations in certain extent.

Oscillospiraceae is referred to as beneficial and butyrate producers, with the ability to produce butyric acid using not only polysaccharides but also glucose (Leth et al., 2023; Van den Abbeele et al., 2023; Wu et al., 2023; Yan et al., 2023; Meng and Shu, 2024). Our study found that the genus of *uncultured_*Oscillospiraceae increased throughout the duration of wound healing in the hydrogel group, suggesting that bacteria and its metabolite butyrate may participate in tissue regeneration in injured skin.

Skin wound healing is a dynamic three-phase process: inflammation, tissue formation, and tissue remodeling (Peña and Martin, 2024). Regulation of inflammation is a key process involved in wound healing which is regulated by short chain fatty acids (SCFAs, mainly butyrate) via activation of kinds of G protein-coupled receptors and inhibition of histone deacetylases (Li et al., 2018; Yao et al., 2022). In addition, aryl hydrocarbon receptor (AHR) is involved in skin wound healing (Ikuta et al., 2009; Chen et al., 2021). However, depending upon its ligand and context, AHR modulation could turn into a “double-edged sword”, resulting in advantageous or harmful effects on skin regeneration (Lozza et al., 2019). Inactivation or loss of AHR accelerates wound closure during the early phase, which corresponds to the inflammatory phase, of wound healing (Carvajal-Gonzalez et al., 2009; Lozza et al., 2019). On the other hand, a recent study implied that lung endothelial AHR prevents lung barrier damage to response for viral infection (Major et al., 2023). Furthermore, Yang and colleagues have demonstrated that SCFAs butyrate could upregulate immune cell AHR expression in the gut (Yang et al., 2020). Therefore, intestinal *uncultured_*Oscillospiraceae may improve skin wound healing by a gut-skin axis (SCFAs/AHR signaling) that needs to be validated in future studies.

Additionally, in our network analysis, genera *Lactobacillus* and *Akkermansia* which are known as SCFAs producers (mainly propionate) (van der Hee and Wells, 2021; Dempsey and Corr, 2022) were found to be keystone microbiota in the hydrogel group. A recent study reported that dietary fiber (used by gut microbiota to yield SCFAs) improves skin wound healing and scar formation through the metabolite-sensing receptor GPR43 (Canesso et al., 2023). These evidences further provide insights into the key role of SCFAs in skin wound healing and benefits of the Sal-hydrogel dressings. Thus, targeted metabolomics is warranted to excavate the SCFAs level in future study.

Understanding microbial interactions is increasingly important due to the diverse range of host-microbe interactions that play a crucial role in maintaining health. An ecological perspective on these interactions and processes can facilitate the development of therapies aimed at mitigating dysbiosis, bacterial infections, and other “microbiota-related” diseases. Thus, we analyzed the bacterial and bacterial interkingdom interactions in gut among the four groups at the initial and final points of wound healing. In the current study, an interesting phenomenon is that the interactions among gut microbiota were enhanced when the wounds were infected by *S. aureus* or not by using our Sal-hydrogel dressings. Generally, microbial interactions enhance environmental fitness and expand ecological niches under perturbations. Our data indicate a complex microbial ecologic community in gut treated by Sal-hydrogel dressings and further demonstrate that the unique function of Sal-hydrogel promotes microbial interactions; in turn, the complex interactions may boost the tissue regeneration and wound healing in injured skin.

Our data shown that use of Sal-hydrogel dressings enhanced gut microbial function activity predicted by PICRUSt2. For example, pathway involved in the biosynthesis of active form of vitamin B1 (thiamine diphosphate) was improved by treated with Sal-hydrogel. It is reported that vitamin B1 could improve outcomes in patients with burn wounds (Saeg et al., 2021). Propanoate production related pathways (pyruvate fermentation to propanoate I) was also enhanced. As mentioned above, propanoate, one of the most abundant SCFAs, may also improve wound healing (Yao et al., 2022).

Additionally, hydrogels are polymeric materials that possess a range of characteristics suitable for meeting the various requirements of an ideal wound dressing, making them promising candidates for wound care (Ribeiro et al., 2024). While standard hydrogels are well-established, salecan’s combination of biocompatibility, intrinsic antibacterial activity, and tunable physical properties positions it as a competitive alternative, particularly for chronic or infected wounds (Qi et al., 2019). Further *in vivo* and clinical studies are needed to validate its superiority over existing options. While this study demonstrates the functional utility of the Sal-hydrogel, its detailed physicochemical characterization (e.g., mechanical properties, porosity, and long-term stability) will be presented in a subsequent work. Here, we prioritized the validation of antimicrobial activity and wound healing to address the primary research objective.

Our study had several limitations. Firstly, since we sacrificed some animals for other sample collections (blood, skins, etc.) the way in which our feces were sampled was not uniform across all times. Difference of gut microbiota in individual level might affect the results. Meanwhile, only male mice were used to conduct this study. Research has demonstrated that sex and sex steroids influence the composition of the gut microbiota and play a role in mediating wound healing (Thomason et al., 2015; Hokanson et al., 2024). The exclusion of females in current research limits our progress in understanding of the impact of gut microbiota on wound healing, particularly with regard to sex differences. Furthermore, our sample size was a little small when considering time point (four mice per group). The limited sample size may affect statistical power and the robustness of our conclusions. Based on Arifin and Zahiruddin, our sample size/group should be five (Arifin and Zahiruddin, 2017). While this number is close to our current sample size, a larger sample size is warranted to further validate our findings regarding microbiota. This study did not employ blinding during treatments and wound assessments, as treatment groups were visually distinguishable. While objective measures were used to reduce bias, future work will implement coding protocols to enable blinding where feasible.

Secondly, the skin microbiota was not included. Wound microbiota can influence each stage of the multifactorial repair process and affect the likelihood of infection. However, the current study aims to investigate changes in gut microbiota. We acknowledge that gut-skin crosstalk is also crucial in skin wound healing. Understanding these crosstalk processes between the gut and the skin reveals numerous possibilities, primarily through the manipulation of the gut microbiome. This may represent therapeutic strategies that could enhance standard treatments for patients with skin injuries, ultimately improving their quality of life. Thirdly, microbial taxonomic resolution (genus-level) and functions characterized by amplicon sequencing were limited in the current study; therefore, a need exists to improve upon this constraint through other technologies like metagenome, metabolomics, and metatranscriptome in future. Fourthly, the direct role of Sal-hydrogel in modulating transdermal signaling remains underexplored, but its material properties likely play a key role in facilitating these processes. Sal-hydrogel’s superior moisture retention, bioadhesion, and porous structure create an optimal wound microenvironment that actively modulates transdermal signaling (Shah et al., 2025). While we did not experimentally isolate the individual contributions of each component, this study focused on the combinatorial effects of the composite Sal-hydrogel (Sal/DA/AgNPs/PVA) in relation to anti-infection and wound healing. Future studies will aim to quantify both their independent and synergistic roles.

Lastly, another restriction is that differences in taxonomic composition (e.g., higher *Lactobacillus* in mice vs Bacteroidales in humans), metabolic function, immune-microbe interactions, and dietary influences limit direct translatability; we targeted bacteria and pathways which might not hold true for the human gut. Despite these disparities, mice remain valuable for mechanistic studies, particularly in germ-free and transgenic models, offering insights into microbial pathways that may inform human research. To bridge findings, results should be validated in humanized mice, organoids, or clinical studies, ensuring relevance before therapeutic applications. Combining mouse experiments with human data and multi-omics approaches will enhance the translational potential. Regardless, our results demonstrated a potential link between gut microbiome, Sal-hydrogel, and wound healing, suggesting that further exploration of this relationship in wound healing for diabetes is worthwhile.

## Conclusion

In summary, significant and rapid perturbations of the gut microbiota response to skin injuries and wound infections, resulting in increased diversity, enhanced correlations, and altered predicted functions. The improved wound healing properties of the Sal-hydrogel dressing linked to high intestinal microbiota diversity and the presence/abundance of the genus *uncultured_*Oscillospiraceae. Our study offers insights into skin wound healing when treated with Sal-hydrogel dressing, suggesting that its effects may be mediated through gut microbiota.

## Data Availability

The data presented in the study are deposited in the Genome Sequence Archive (GSA) repository in National Genomics Data Center, China National Center for Bioinformation / Beijing Institute of Genomics, Chinese Academy of Sciences, accession number CRA015749.
