# Supplementary material for: Dynamic male mouse gut microbiota signature linked to improved wound healing of a novel salecan hydrogel dressing
Source: Front Bioeng Biotechnol. 2025 Aug 21;13:1584976. doi: 10.3389/fbioe.2025.1584976 (PMC12409744; doi:10.3389/fbioe.2025.1584976)
Supplement: Supplementary file 1 [file DataSheet1.docx]

Supplementary Materials

# Supplementary Figures


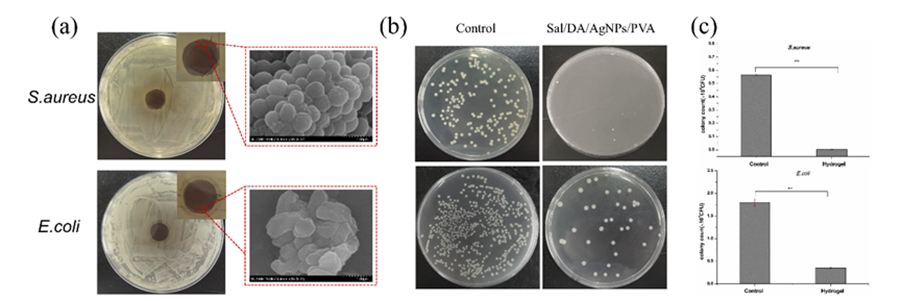


**Fig. S1.** **Antibacterial activity of hydrogel samples, against *E. coli* and *S. aureus*.** (a) Antibacterial circle and biological SEM, (b) Diluted coating plate, (c) Corresponding statistical data of *S. aureus* and *E. coli* colonies, **p* < 0.05, ***p* < 0.01.

**
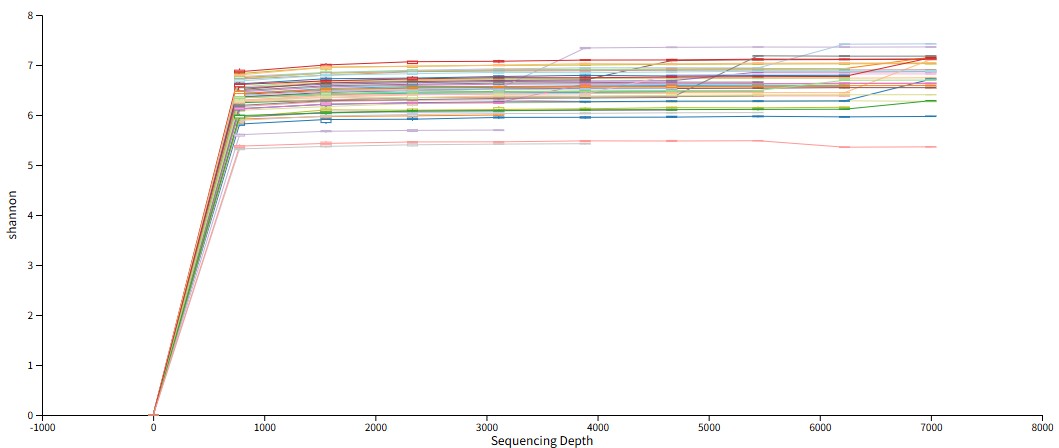
**

**Fig. S2. Rarefaction curves of all samples.** The different color of lines represents different samples. The curves of Shannon diversity reached saturation in all samples suggesting the sequencing depth was sufficient.


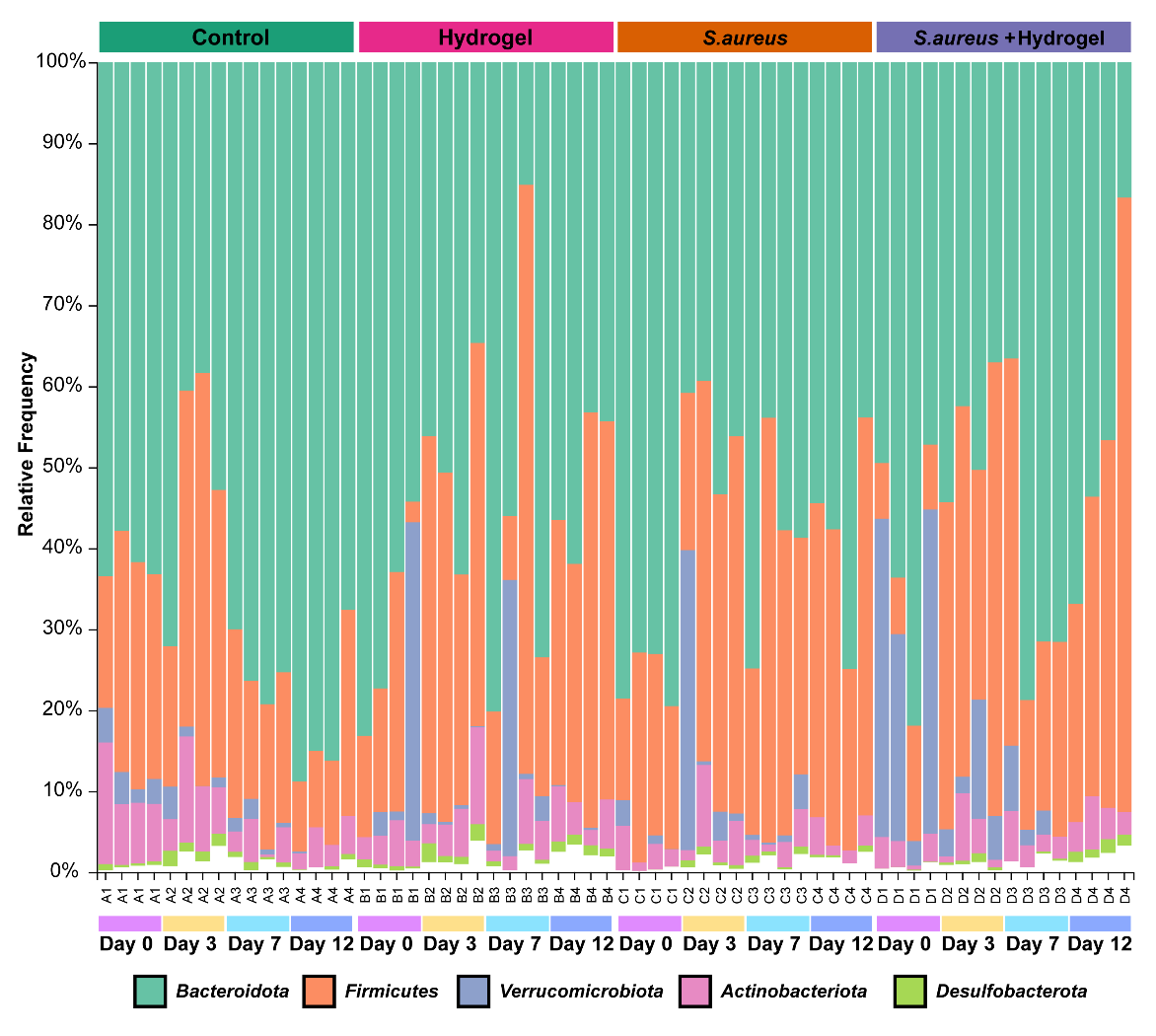


**Fig. S3. Bar plot depicted the bacterial relative abundance at the phylum level.** Top five phyla were shown.


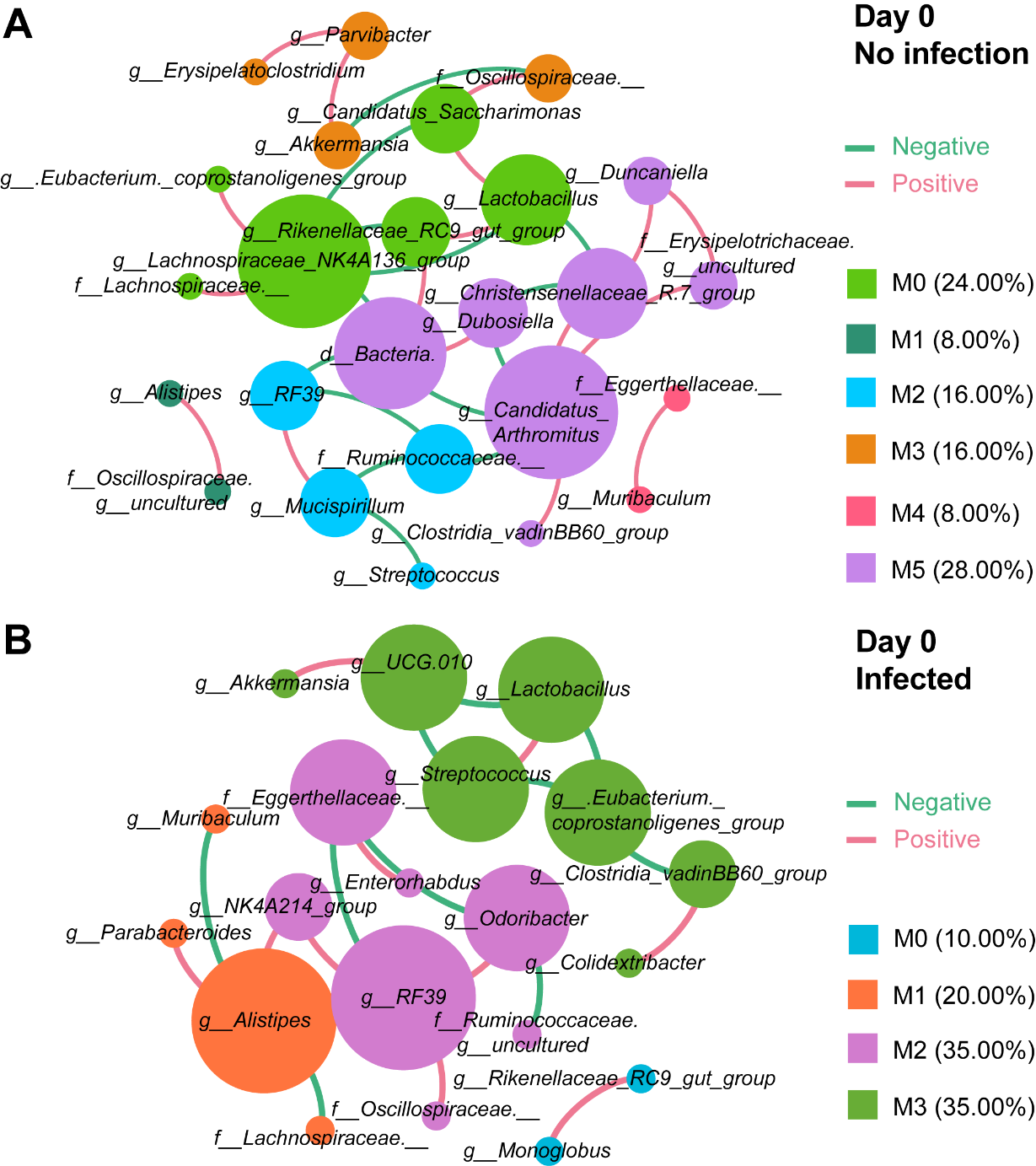


**Fig. S4.** **Co-occurrence networks of the bacterial-bacterial correlations in gut at first day.** The data of the first day (day 0) of control and hydrogel groups was merged as control for uninfected group (**A**), S. aureus and S. aureus and hydrogel groups as control for infected group (**B**). Node size was presented by its degree. Edge colors presented interaction between two nodes. Red edges represent positive correlation, and green represent negative correlation. “M” represented the module of the network.

# Supplementary Tables

**Table S1 SparCC correlation network in blank group at end point**

| **Source** | **Interaction** | **Target** | **Correlation** | ***p* value** |
| --- | --- | --- | --- | --- |
| *g__Odoribacter* | positive | *g__Monoglobus* | 0.657552 | 0.05 |
| *g__Duncaniella* | negative | *g__Mucispirillum* | -0.71713 | 0.03 |
| *g__Enterorhabdus* | positive | *g__Candidatus_Saccharimonas* | 0.668252 | 0.05 |
| *g__Duncaniella* | positive | *g__Parabacteroides* | 0.838425 | 0.01 |
| *g__Mucispirillum* | negative | *g__Rikenellaceae_RC9_gut_group* | -0.65922 | 0.04 |
| *g__Parabacteroides* | positive | *g__Rikenellaceae_RC9_gut_group* | 0.745357 | 0.04 |
| *g__Alistipes* | positive | *g__Muribaculum* | 0.793241 | 0.05 |
| *g__Rikenellaceae_RC9_gut_group* | positive | *g__Muribaculum* | 0.874339 | 0 |
| *g__Mucispirillum* | positive | *f__Oscillospiraceae.__* | 0.785564 | 0.03 |
| *g__Lachnospiraceae_NK4A136_group* | positive | *g__Clostridia_vadinBB60_group* | 0.726389 | 0 |
| *g__Desulfovibrio* | positive | *g__Clostridia_vadinBB60_group* | 0.621209 | 0.04 |
| *g__Muribaculum* | positive | *g__ASF356* | 0.568576 | 0.04 |
| *g__NK4A214_group* | positive | *g__Colidextribacter* | 0.65886 | 0.01 |
| *g__Lachnospiraceae_NK4A136_group* | negative | *g__Bacteroides* | -0.7678 | 0 |
| *g__Mucispirillum* | negative | *o__Bacteroidales.__.__* | -0.49082 | 0.05 |
| *g__Rikenellaceae_RC9_gut_group* | positive | *o__Bacteroidales.__.__* | 0.503163 | 0.05 |

**Table S2 SparCC correlation network in hydrogel group at end point**

| **Source** | **Interaction** | **Target** | **Correlation** | ***p* value** |
| --- | --- | --- | --- | --- |
| *g__Alistipes* | positive | *g__Odoribacter* | 0.800831 | 0.04 |
| *g__Lactobacillus* | positive | *g__Enterorhabdus* | 0.737854 | 0.01 |
| *g__Lactobacillus* | negative | *f__Oscillospiraceae.g__uncultured* | -0.75992 | 0.02 |
| *g__Akkermansia* | negative | *g__Mucispirillum* | -0.71176 | 0.02 |
| *g__Odoribacter* | negative | *g__Candidatus_Saccharimonas* | -0.7566 | 0.04 |
| *g__RF39* | positive | *g__Candidatus_Saccharimonas* | 0.638339 | 0.05 |
| *g__Duncaniella* | positive | *g__Parabacteroides* | 0.708791 | 0.04 |
| *g__Alistipes* | positive | *g__Rikenellaceae_RC9_gut_group* | 0.89532 | 0 |
| *g__Odoribacter* | positive | *g__Rikenellaceae_RC9_gut_group* | 0.695455 | 0.03 |
| *g__Parabacteroides* | positive | *g__Rikenellaceae_RC9_gut_group* | 0.630736 | 0.04 |
| *g__Akkermansia* | negative | *f__Desulfovibrionaceae.g__uncultured* | -0.73546 | 0.04 |
| *g__Mucispirillum* | positive | *f__Desulfovibrionaceae.g__uncultured* | 0.798973 | 0.02 |
| *g__Duncaniella* | negative | *g__Roseburia* | -0.74097 | 0.01 |
| *g__Duncaniella* | positive | *g__Muribaculum* | 0.969062 | 0 |
| *g__Roseburia* | negative | *g__Muribaculum* | -0.7019 | 0.02 |
| *g__Lachnospiraceae_NK4A136_group* | positive | *f__Oscillospiraceae.__* | 0.748668 | 0.03 |
| *g__Roseburia* | negative | *f__Ruminococcaceae.__* | -0.74325 | 0 |
| *g__Gastranaerophilales* | positive | *f__Ruminococcaceae.g__uncultured* | 0.61121 | 0.02 |
| *g__Roseburia* | positive | *g__Incertae_Sedis* | 0.716217 | 0.02 |
| *g__Parabacteroides* | positive | *g__Clostridia_vadinBB60_group* | 0.698868 | 0.05 |
| *g__Alistipes* | negative | *g__Parvibacter* | -0.7028 | 0.02 |
| *g__Rikenellaceae_RC9_gut_group* | negative | *g__Parvibacter* | -0.82201 | 0.01 |
| *g__Odoribacter* | positive | *g__Ruminococcaceae* | 0.794784 | 0 |
| *g__Lachnospiraceae_NK4A136_group* | positive | *g__Colidextribacter* | 0.604213 | 0.03 |
| *g__Monoglobus* | negative | *g__Colidextribacter* | -0.87469 | 0 |
| *g__Enterorhabdus* | negative | *c__Clostridia.__.__.__* | -0.61757 | 0.01 |
| *g__Gastranaerophilales* | positive | *c__Clostridia.__.__.__* | 0.501537 | 0.05 |
| *g__Lactobacillus* | negative | *g__Oscillibacter* | -0.60265 | 0.05 |
| *g__Duncaniella* | negative | *f__Lachnospiraceae.g__Blautia* | -0.66061 | 0.03 |
| *g__Odoribacter* | negative | *f__Lachnospiraceae.g__Blautia* | -0.60088 | 0.02 |
| *g__Erysipelatoclostridium* | positive | *f__Lachnospiraceae.g__Blautia* | 0.661132 | 0.03 |
| *g__Muribaculum* | negative | *f__Lachnospiraceae.g__Blautia* | -0.6095 | 0.05 |
| *g__Duncaniella* | positive | *d__Bacteria.__.__.__.__.__* | 0.613879 | 0.03 |
| *g__Akkermansia* | positive | *d__Bacteria.__.__.__.__.__* | 0.673527 | 0.04 |
| *g__Alistipes* | positive | *d__Bacteria.__.__.__.__.__* | 0.779572 | 0 |
| *g__Odoribacter* | positive | *d__Bacteria.__.__.__.__.__* | 0.776533 | 0.02 |
| *g__Mucispirillum* | negative | *d__Bacteria.__.__.__.__.__* | -0.64251 | 0.02 |
| *g__RF39* | negative | *d__Bacteria.__.__.__.__.__* | -0.54794 | 0.05 |
| *g__Candidatus_Saccharimonas* | negative | *d__Bacteria.__.__.__.__.__* | -0.77732 | 0 |
| *g__Rikenellaceae_RC9_gut_group* | positive | *d__Bacteria.__.__.__.__.__* | 0.682656 | 0.01 |
| *g__Muribaculum* | positive | *d__Bacteria.__.__.__.__.__* | 0.627059 | 0.04 |

**Table S3 SparCC correlation network in *S. aureus* group at end point**

| **Source** | **Interaction** | **Target** | **Correlation** | ***p* value** |
| --- | --- | --- | --- | --- |
| *g__Akkermansia* | negative | *g__Lachnospiraceae_NK4A136_group* | -0.75198 | 0 |
| *g__Lactobacillus* | positive | *g__Odoribacter* | 0.761015 | 0.02 |
| *g__Enterorhabdus* | positive | *g__Monoglobus* | 0.708536 | 0.02 |
| *g__Duncaniella* | negative | *f__Lachnospiraceae.__* | -0.73563 | 0.01 |
| *g__Monoglobus* | negative | *f__Lachnospiraceae.__* | -0.60803 | 0.04 |
| *g__Akkermansia* | positive | *g__Prevotellaceae_UCG.001* | 0.856008 | 0 |
| *g__Lachnospiraceae_NK4A136_group* | negative | *g__Prevotellaceae_UCG.001* | -0.77243 | 0.01 |
| *g__Odoribacter* | negative | *g__Mucispirillum* | -0.68816 | 0.02 |
| *g__Akkermansia* | negative | *g__RF39* | -0.69424 | 0.02 |
| *g__Akkermansia* | negative | *g__Candidatus_Saccharimonas* | -0.71513 | 0.01 |
| *g__Alistipes* | negative | *g__Gastranaerophilales* | -0.68633 | 0.04 |
| *g__Monoglobus* | positive | *g__Erysipelatoclostridium* | 0.607388 | 0.02 |
| *g__Lactobacillus* | negative | *f__Desulfovibrionaceae.g__uncultured* | -0.76608 | 0.02 |
| *g__Odoribacter* | negative | *f__Desulfovibrionaceae.g__uncultured* | -0.74619 | 0.01 |
| *g__Mucispirillum* | positive | *g__NK4A214_group* | 0.74237 | 0.04 |
| *g__Akkermansia* | positive | *g__Staphylococcus* | 0.725183 | 0.02 |
| *g__Lachnospiraceae_NK4A136_group* | negative | *g__Staphylococcus* | -0.71052 | 0.05 |
| *g__Duncaniella* | positive | *g__Muribaculum* | 0.830279 | 0.02 |
| *g__Mucispirillum* | positive | *f__Oscillospiraceae.__* | 0.771063 | 0 |
| *g__NK4A214_group* | positive | *f__Oscillospiraceae.__* | 0.776449 | 0 |
| *g__Odoribacter* | negative | *f__Ruminococcaceae.g__uncultured* | -0.68229 | 0.03 |
| *g__Muribaculum* | negative | *f__Ruminococcaceae.g__uncultured* | -0.6668 | 0.02 |
| *g__RF39* | positive | *g__Desulfovibrio* | 0.775155 | 0 |
| *g__Akkermansia* | negative | *g__Streptococcus* | -0.71843 | 0.04 |
| *g__Odoribacter* | positive | *g__Streptococcus* | 0.664043 | 0.02 |
| *g__Candidatus_Saccharimonas* | positive | *g__Streptococcus* | 0.799148 | 0 |
| *g__Staphylococcus* | negative | *g__Streptococcus* | -0.55892 | 0.05 |
| *g__Akkermansia* | negative | *g__Clostridia_vadinBB60_group* | -0.75295 | 0.01 |
| *g__Lachnospiraceae_NK4A136_group* | positive | *g__Clostridia_vadinBB60_group* | 0.687166 | 0.04 |
| *g__Staphylococcus* | negative | *g__Clostridia_vadinBB60_group* | -0.58448 | 0.04 |
| *g__NK4A214_group* | negative | *g__ASF356* | -0.55457 | 0.05 |
| *g__Lactobacillus* | positive | *g__Alloprevotella* | 0.784921 | 0.01 |
| *g__Odoribacter* | positive | *g__Alloprevotella* | 0.724364 | 0.01 |
| *g__Mucispirillum* | negative | *g__Alloprevotella* | -0.65345 | 0.03 |
| *g__ASF356* | positive | *g__Alloprevotella* | 0.610218 | 0.05 |
| *g__Odoribacter* | negative | *g__Anaerotruncus* | -0.69302 | 0.04 |
| *g__Mucispirillum* | positive | *g__Anaerotruncus* | 0.674728 | 0.02 |
| *g__Alloprevotella* | negative | *g__Anaerotruncus* | -0.75154 | 0.02 |
| *g__Akkermansia* | positive | *g__Ruminococcaceae* | 0.66775 | 0.05 |
| *g__Staphylococcus* | positive | *g__Ruminococcaceae* | 0.677574 | 0.04 |
| *g__Alistipes* | positive | *g__Colidextribacter* | 0.626466 | 0.04 |
| *g__RF39* | negative | *d__Bacteria.__.__.__.__.__* | -0.56789 | 0.03 |
| *g__Roseburia* | negative | *d__Bacteria.__.__.__.__.__* | -0.60091 | 0.04 |
| *g__Colidextribacter* | positive | *d__Bacteria.__.__.__.__.__* | 0.557769 | 0.03 |

**Table S4 SparCC correlation network in *S. aureus* and hydrogel group at end point**

| **Source** | **Interaction** | **Target** | **Correlation** | ***p* value** |
| --- | --- | --- | --- | --- |
| *g__Alistipes* | negative | *g__Lachnospiraceae_NK4A136_group* | -0.83003 | 0 |
| *g__Duncaniella* | positive | *g__Odoribacter* | 0.842827 | 0.02 |
| *g__Lactobacillus* | positive | *g__Odoribacter* | 0.63631 | 0.03 |
| *g__Duncaniella* | positive | *g__Enterorhabdus* | 0.716292 | 0.05 |
| *g__Odoribacter* | positive | *g__Enterorhabdus* | 0.792244 | 0.03 |
| *g__Lachnospiraceae_NK4A136_group* | positive | *f__Lachnospiraceae.g__uncultured* | 0.669343 | 0.02 |
| *g__Duncaniella* | negative | *f__Oscillospiraceae.g__uncultured* | -0.74669 | 0.04 |
| *g__Odoribacter* | negative | *f__Oscillospiraceae.g__uncultured* | -0.86657 | 0 |
| *g__Enterorhabdus* | negative | *f__Oscillospiraceae.g__uncultured* | -0.82669 | 0 |
| *g__Monoglobus* | positive | *g__Mucispirillum* | 0.780402 | 0.02 |
| *g__Lactobacillus* | positive | *g__Candidatus_Saccharimonas* | 0.712968 | 0.03 |
| *g__Alistipes* | positive | *g__Candidatus_Saccharimonas* | 0.64596 | 0.05 |
| *g__Odoribacter* | positive | *g__Candidatus_Saccharimonas* | 0.728744 | 0.03 |
| *g__Lachnospiraceae_NK4A136_group* | positive | *g__Gastranaerophilales* | 0.792581 | 0 |
| *g__Monoglobus* | positive | *g__Gastranaerophilales* | 0.692859 | 0.04 |
| *g__Duncaniella* | positive | *g__Parabacteroides* | 0.801101 | 0.02 |
| *g__Candidatus_Arthromitus* | negative | *g__Parabacteroides* | -0.74783 | 0 |
| *g__Duncaniella* | positive | *g__Rikenellaceae_RC9_gut_group* | 0.947408 | 0 |
| *g__Odoribacter* | positive | *g__Rikenellaceae_RC9_gut_group* | 0.869312 | 0 |
| *g__Parabacteroides* | positive | *g__Rikenellaceae_RC9_gut_group* | 0.791022 | 0.01 |
| *g__Candidatus_Arthromitus* | positive | *g__Roseburia* | 0.735288 | 0.01 |
| *g__Duncaniella* | positive | *g__Muribaculum* | 0.755953 | 0.02 |
| *g__Odoribacter* | positive | *g__Muribaculum* | 0.714705 | 0.05 |
| *g__Rikenellaceae_RC9_gut_group* | positive | *g__Muribaculum* | 0.867792 | 0.01 |
| *g__Lactobacillus* | negative | *g__Ruminococcus* | -0.82618 | 0.03 |
| *g__Odoribacter* | negative | *f__Ruminococcaceae.g__uncultured* | -0.68505 | 0.01 |
| *g__Staphylococcus* | negative | *f__Ruminococcaceae.g__uncultured* | -0.68354 | 0.03 |
| *g__Muribaculum* | negative | *f__Ruminococcaceae.g__uncultured* | -0.66839 | 0.05 |
| *g__Duncaniella* | negative | *g__Incertae_Sedis* | -0.66823 | 0.03 |
| *g__RF39* | positive | *g__Incertae_Sedis* | 0.697852 | 0.05 |
| *g__Parabacteroides* | negative | *g__Incertae_Sedis* | -0.65992 | 0.02 |
| *g__Rikenellaceae_RC9_gut_group* | negative | *g__Incertae_Sedis* | -0.67095 | 0.03 |
| *g__Odoribacter* | negative | *g__Desulfovibrio* | -0.72575 | 0.04 |
| *g__Enterorhabdus* | negative | *g__Desulfovibrio* | -0.66837 | 0.03 |
| *g__Mucispirillum* | positive | *g__Desulfovibrio* | 0.654853 | 0.01 |
| *g__Candidatus_Saccharimonas* | negative | *g__Desulfovibrio* | -0.71489 | 0.04 |
| *g__Rikenellaceae_RC9_gut_group* | negative | *g__Desulfovibrio* | -0.6415 | 0.02 |
| *g__Muribaculum* | negative | *g__Desulfovibrio* | -0.75648 | 0 |
| *g__RF39* | positive | *f__Erysipelotrichaceae.g__uncultured* | 0.701281 | 0.04 |
| *g__Staphylococcus* | positive | *f__Erysipelotrichaceae.g__uncultured* | 0.672101 | 0.01 |
| *g__RF39* | positive | *g__Streptococcus* | 0.817691 | 0 |
| *g__Incertae_Sedis* | positive | *g__Streptococcus* | 0.734591 | 0.04 |
| *g__Odoribacter* | negative | *g__Clostridia_vadinBB60_group* | -0.76615 | 0.01 |
| *g__Candidatus_Saccharimonas* | negative | *g__Clostridia_vadinBB60_group* | -0.68765 | 0.05 |
| *g__Rikenellaceae_RC9_gut_group* | negative | *g__Clostridia_vadinBB60_group* | -0.70281 | 0.03 |
| *g__Muribaculum* | negative | *g__Clostridia_vadinBB60_group* | -0.65619 | 0.05 |
| *g__Lachnospiraceae_NK4A136_group* | positive | *g__ASF356* | 0.683503 | 0.05 |
| *g__Odoribacter* | negative | *g__ASF356* | -0.702 | 0 |
| *g__Monoglobus* | positive | *g__ASF356* | 0.726461 | 0.05 |
| *g__Mucispirillum* | positive | *g__ASF356* | 0.867015 | 0 |
| *g__Candidatus_Saccharimonas* | negative | *g__ASF356* | -0.72494 | 0.01 |
| *g__Desulfovibrio* | positive | *g__ASF356* | 0.731389 | 0.03 |
| *g__Duncaniella* | positive | *c__Clostridia.__.__.__* | 0.641241 | 0.02 |
| *g__Monoglobus* | negative | *c__Clostridia.__.__.__* | -0.75529 | 0 |
| *g__Mucispirillum* | negative | *c__Clostridia.__.__.__* | -0.63732 | 0.03 |
| *g__RF39* | negative | *c__Clostridia.__.__.__* | -0.6144 | 0.02 |
| *g__Parabacteroides* | positive | *c__Clostridia.__.__.__* | 0.645588 | 0.02 |
| *g__Streptococcus* | negative | *c__Clostridia.__.__.__* | -0.69567 | 0.01 |
| *g__Parabacteroides* | positive | *g__Bacteroides* | 0.727676 | 0.01 |
| *g__Rikenellaceae_RC9_gut_group* | positive | *g__Bacteroides* | 0.670262 | 0.05 |
| *g__Incertae_Sedis* | negative | *g__Bacteroides* | -0.67377 | 0.02 |
| *g__Duncaniella* | positive | *o__Bacteroidales.__.__* | 0.692182 | 0.02 |
| *g__Alistipes* | positive | *o__Bacteroidales.__.__* | 0.515867 | 0.05 |
| *g__Lachnospiraceae_NK4A136_group* | negative | *o__Bacteroidales.__.__* | -0.71964 | 0 |
| *g__Odoribacter* | positive | *o__Bacteroidales.__.__* | 0.605817 | 0.01 |
| *g__Monoglobus* | negative | *o__Bacteroidales.__.__* | -0.68994 | 0.01 |
| *g__Mucispirillum* | negative | *o__Bacteroidales.__.__* | -0.62494 | 0.03 |
| *g__Gastranaerophilales* | negative | *o__Bacteroidales.__.__* | -0.58292 | 0.02 |
| *g__Parabacteroides* | positive | *o__Bacteroidales.__.__* | 0.732188 | 0 |
| *g__ASF356* | negative | *o__Bacteroidales.__.__* | -0.62418 | 0.02 |
| *g__Parabacteroides* | positive | *d__Bacteria.__.__.__.__.__* | 0.630648 | 0.04 |
| *g__Bacteroides* | positive | *d__Bacteria.__.__.__.__.__* | 0.627709 | 0.04 |

**Table S5 SparCC correlation network in uninfected group at first day**

| **Source** | **Interaction** | **Target** | **Correlation** | ***p* value** |
| --- | --- | --- | --- | --- |
| *g__Lactobacillus* | negative | *g__Lachnospiraceae_NK4A136_group* | -0.69511 | 0.05 |
| *g__Lachnospiraceae_NK4A136_group* | positive | *f__Lachnospiraceae.__* | 0.821735 | 0.02 |
| *g__Alistipes* | positive | *f__Oscillospiraceae.g__uncultured* | 0.740342 | 0.03 |
| *g__Mucispirillum* | positive | *g__RF39* | 0.72762 | 0.02 |
| *g__Lactobacillus* | positive | *g__Candidatus_Saccharimonas* | 0.759629 | 0.01 |
| *g__Lachnospiraceae_NK4A136_group* | negative | *g__Candidatus_Saccharimonas* | -0.69993 | 0.04 |
| *g__Lactobacillus* | positive | *g__Rikenellaceae_RC9_gut_group* | 0.719192 | 0.03 |
| *g__Lachnospiraceae_NK4A136_group* | negative | *g__Rikenellaceae_RC9_gut_group* | -0.82703 | 0 |
| *g__Akkermansia* | negative | *f__Oscillospiraceae.__* | -0.70517 | 0.03 |
| *g__Candidatus_Saccharimonas* | positive | *f__Oscillospiraceae.__* | 0.746987 | 0.02 |
| *g__Candidatus_Arthromitus* | negative | *f__Ruminococcaceae.__* | -0.60346 | 0.04 |
| *g__Mucispirillum* | negative | *f__Ruminococcaceae.__* | -0.60761 | 0 |
| *g__RF39* | negative | *f__Ruminococcaceae.__* | -0.60181 | 0.05 |
| *g__Duncaniella* | positive | *f__Erysipelotrichaceae.g__uncultured* | 0.677451 | 0.04 |
| *g__Candidatus_Arthromitus* | positive | *f__Erysipelotrichaceae.g__uncultured* | 0.626794 | 0.03 |
| *g__Mucispirillum* | negative | *g__Streptococcus* | -0.59814 | 0.02 |
| *g__Candidatus_Arthromitus* | positive | *g__Clostridia_vadinBB60_group* | 0.741952 | 0.02 |
| *g__Akkermansia* | positive | *g__Parvibacter* | 0.597014 | 0.05 |
| *g__Erysipelatoclostridium* | positive | *g__Parvibacter* | 0.808465 | 0 |
| *g__Candidatus_Arthromitus* | negative | *g__Dubosiella* | -0.73822 | 0.01 |
| *g__Muribaculum* | positive | *f__Eggerthellaceae.__* | 0.662047 | 0.04 |
| *g__Lachnospiraceae_NK4A136_group* | positive | *g__.Eubacterium._coprostanoligenes_group* | 0.692176 | 0.01 |
| *g__Duncaniella* | positive | *g__Christensenellaceae_R.7_group* | 0.77559 | 0 |
| *g__Lactobacillus* | negative | *g__Christensenellaceae_R.7_group* | -0.66111 | 0.01 |
| *g__Candidatus_Arthromitus* | positive | *g__Christensenellaceae_R.7_group* | 0.692397 | 0.02 |
| *g__Dubosiella* | negative | *g__Christensenellaceae_R.7_group* | -0.61408 | 0.01 |
| *g__Lachnospiraceae_NK4A136_group* | negative | *d__Bacteria.__.__.__.__.__* | -0.62981 | 0.02 |
| *g__Candidatus_Arthromitus* | negative | *d__Bacteria.__.__.__.__.__* | -0.70148 | 0.02 |
| *g__RF39* | negative | *d__Bacteria.__.__.__.__.__* | -0.59555 | 0.04 |
| *g__Rikenellaceae_RC9_gut_group* | positive | *d__Bacteria.__.__.__.__.__* | 0.64063 | 0 |
| *g__Dubosiella* | positive | *d__Bacteria.__.__.__.__.__* | 0.654229 | 0 |

**Table S6 SparCC correlation network in infected group at first day**

| **Source** | **Interaction** | **Target** | **Correlation** | ***P* value** |
| --- | --- | --- | --- | --- |
| *g__Alistipes* | negative | *f__Lachnospiraceae.__* | -0.76813 | 0.02 |
| *g__Odoribacter* | positive | *g__RF39* | 0.850914 | 0 |
| *g__Alistipes* | positive | *g__Parabacteroides* | 0.610211 | 0.05 |
| *g__Monoglobus* | positive | *g__Rikenellaceae_RC9_gut_group* | 0.814836 | 0.05 |
| *g__Alistipes* | positive | *g__NK4A214_group* | 0.587486 | 0.05 |
| *g__RF39* | positive | *g__NK4A214_group* | 0.630207 | 0.02 |
| *g__Alistipes* | negative | *g__Muribaculum* | -0.75922 | 0.03 |
| *g__RF39* | positive | *f__Oscillospiraceae.__* | 0.670478 | 0.05 |
| *g__Odoribacter* | negative | *f__Ruminococcaceae.g__uncultured* | -0.64619 | 0.04 |
| *g__Lactobacillus* | positive | *g__Streptococcus* | 0.82437 | 0 |
| *g__Odoribacter* | negative | *f__Eggerthellaceae.__* | -0.65589 | 0.03 |
| *g__Enterorhabdus* | positive | *f__Eggerthellaceae.__* | 0.604237 | 0.03 |
| *g__RF39* | negative | *f__Eggerthellaceae.__* | -0.72616 | 0.01 |
| *g__Clostridia_vadinBB60_group* | positive | *g__Colidextribacter* | 0.643811 | 0.03 |
| *g__Lactobacillus* | negative | *g__.Eubacterium._coprostanoligenes_group* | -0.66262 | 0.05 |
| *g__Streptococcus* | negative | *g__.Eubacterium._coprostanoligenes_group* | -0.66981 | 0.01 |
| *g__Clostridia_vadinBB60_group* | negative | *g__.Eubacterium._coprostanoligenes_group* | -0.6017 | 0.04 |
| *g__Lactobacillus* | negative | *g__UCG.010* | -0.72134 | 0 |
| *g__Akkermansia* | positive | *g__UCG.010* | 0.691093 | 0.04 |
| *g__Streptococcus* | negative | *g__UCG.010* | -0.72384 | 0.02 |

**Table S7 Significantly changed predicted pathways at day 0**

| **Pathways** | **mean_Control** | **mean_Hydrogel** | **mean_*S.aureus*** | **mean_*S.aureus*+Hydrogel** | **sd_Control** | **sd_Hydrogel** | **sd_*S.aureus*** | **sd_*S.aureus*+Hydrogel** | **se_Control** | **se_Hydrogel** | **se_*S.aureus*** | **se_*S.aureus*+Hydrogel** | ***P* value adjusted** | **sign** |
| --- | --- | --- | --- | --- | --- | --- | --- | --- | --- | --- | --- | --- | --- | --- |
| CMP-3-deoxy-D-manno-octulosonate biosynthesis I | 978.8400347 | 2142.920865 | 1052.529944 | 1743.439044 | 194.419059 | 1087.13238 | 88.2059008 | 64.04414811 | 97.2095295 | 543.566191 | 44.1029504 | 32.02207406 | 0.043227 | * |
| glyoxylate cycle | 2.992931155 | 60.71731984 | 0.997795582 | 40.14267635 | 3.45593923 | 17.9922597 | 1.99559116 | 31.61378593 | 1.72796961 | 8.99612985 | 0.99779558 | 15.80689296 | 0.043227 | * |
| inosine-5'-phosphate biosynthesis III | 1571.298377 | 261.5370403 | 1286.559051 | 134.1446263 | 525.901583 | 183.06339 | 638.597533 | 89.85764341 | 262.950791 | 91.5316952 | 319.298767 | 44.92882171 | 0.043227 | * |
| Kdo transfer to lipid IVA III (Chlamydia) | 971.0775 | 2130.37 | 1051.815 | 1736.14 | 196.939356 | 1091.20826 | 89.0701183 | 65.24081902 | 98.4696779 | 545.604128 | 44.5350591 | 32.62040951 | 0.043227 | * |
| lipid IVA biosynthesis | 1065.393628 | 2353.009182 | 1162.703149 | 1880.415968 | 209.239791 | 1235.13262 | 90.8212106 | 80.89766767 | 104.619895 | 617.566308 | 45.4106053 | 40.44883384 | 0.043227 | * |
| mevalonate pathway I | 433.543106 | 58.43741765 | 375.8782467 | 23.21328335 | 136.017751 | 37.8339674 | 178.289505 | 29.28036292 | 68.0088757 | 18.9169837 | 89.1447525 | 14.64018146 | 0.043227 | * |
| phosphopantothenate biosynthesis I | 1290.540716 | 2487.826131 | 1292.586321 | 1958.955015 | 271.668801 | 1060.88117 | 140.669149 | 73.67511472 | 135.834401 | 530.440587 | 70.3345747 | 36.83755736 | 0.043227 | * |
| preQ0 biosynthesis | 1074.13711 | 1666.639737 | 1075.807764 | 1778.388606 | 162.816773 | 183.996592 | 86.8429822 | 74.78816259 | 81.4083865 | 91.9982959 | 43.4214911 | 37.3940813 | 0.043227 | * |
| pyruvate fermentation to propanoate I | 1555.726334 | 3121.10109 | 1657.30985 | 2526.123419 | 303.604418 | 1263.76441 | 131.667392 | 87.04764169 | 151.802209 | 631.882204 | 65.8336959 | 43.52382085 | 0.043227 | * |
| queuosine biosynthesis | 1376.654597 | 2086.435165 | 1438.304029 | 2067.903305 | 241.817102 | 151.181152 | 210.480531 | 114.7519891 | 120.908551 | 75.5905758 | 105.240266 | 57.37599453 | 0.043227 | * |
| superpathway of geranylgeranyldiphosphate biosynthesis I (via mevalonate) | 570.8599769 | 82.48239198 | 493.764015 | 32.83456397 | 175.362804 | 52.767852 | 221.71341 | 41.36011519 | 87.681402 | 26.383926 | 110.856705 | 20.6800576 | 0.043227 | * |
| superpathway of glycerol degradation to 1,3-propanediol | 60.15678754 | 0 | 60.73665938 | 1.467871853 | 21.0835833 | 0 | 46.6302338 | 2.935743705 | 10.5417916 | 0 | 23.3151169 | 1.467871853 | 0.043227 | * |
| superpathway of glycol metabolism and degradation | 2.774706831 | 37.39336409 | 0.919516817 | 23.92107845 | 3.20626672 | 18.9933668 | 1.83903363 | 18.7717051 | 1.60313336 | 9.49668338 | 0.91951682 | 9.38585255 | 0.043227 | * |
| superpathway of sulfur oxidation (Acidianus ambivalens) | 71.665 | 104.755 | 0 | 8.42 | 29.9407565 | 63.1236889 | 0 | 9.768602766 | 14.9703783 | 31.5618444 | 0 | 4.884301383 | 0.043227 | * |
| superpathway of tetrahydrofolate biosynthesis | 1064.757855 | 1889.635219 | 787.7643769 | 1675.556088 | 223.455184 | 681.040821 | 239.266732 | 185.673471 | 111.727592 | 340.52041 | 119.633366 | 92.83673549 | 0.043227 | * |
| superpathway of tetrahydrofolate biosynthesis and salvage | 1312.39605 | 2217.717735 | 1004.733408 | 1906.18244 | 258.403582 | 748.457027 | 274.461839 | 126.4355011 | 129.201791 | 374.228514 | 137.23092 | 63.21775054 | 0.043227 | * |
| superpathway of thiamin diphosphate biosynthesis I | 1423.75212 | 2335.569513 | 1426.454995 | 2273.183576 | 280.369655 | 272.901391 | 163.493797 | 150.9974326 | 140.184827 | 136.450695 | 81.7468986 | 75.49871631 | 0.043227 | * |
| thiamin salvage II | 1479.749159 | 2376.580965 | 1435.902281 | 2256.342212 | 342.732724 | 291.895181 | 138.547446 | 133.6724243 | 171.366362 | 145.94759 | 69.273723 | 66.83621214 | 0.043227 | * |
| thiazole biosynthesis I (E. coli) | 1245.243152 | 1958.12559 | 1333.025146 | 2079.334207 | 257.034468 | 263.045464 | 235.157246 | 225.6892723 | 128.517234 | 131.522732 | 117.578623 | 112.8446362 | 0.043227 | * |
| tRNA processing | 55.20101453 | 109.9476344 | 0.748223474 | 35.66568231 | 19.7121951 | 49.1422216 | 1.49644695 | 22.14483447 | 9.85609755 | 24.5711108 | 0.74822347 | 11.07241723 | 0.043227 | * |
| 1,4-dihydroxy-6-naphthoate biosynthesis I | 196.125 | 288.0126324 | 59.34656977 | 115.5163565 | 125.299225 | 201.424957 | 19.3471447 | 35.41322167 | 62.6496126 | 100.712479 | 9.67357236 | 17.70661083 | 0.043227 | * |
| 2-methylcitrate cycle I | 0 | 6.151124258 | 1.388888889 | 9.503011102 | 0 | 3.65042616 | 2.77777778 | 9.052072895 | 0 | 1.82521308 | 1.38888889 | 4.526036448 | 0.048717 | * |
| 2-methylcitrate cycle II | 0 | 6.27993921 | 1.428571429 | 9.559742647 | 0 | 3.57889234 | 2.85714286 | 9.025776664 | 0 | 1.78944617 | 1.42857143 | 4.512888332 | 0.048717 | * |
| 6-hydroxymethyl-dihydropterin diphosphate biosynthesis I | 1249.835654 | 2471.247325 | 1385.131146 | 1664.11303 | 212.496435 | 1093.15518 | 139.103289 | 335.5261467 | 106.248218 | 546.57759 | 69.5516447 | 167.7630733 | 0.043227 | * |
| 6-hydroxymethyl-dihydropterin diphosphate biosynthesis III (Chlamydia) | 1232.750216 | 2461.17364 | 1374.599093 | 1668.528678 | 238.719395 | 1109.32984 | 164.905966 | 382.591677 | 119.359698 | 554.66492 | 82.4529828 | 191.2958385 | 0.043227 | * |
| ADP-L-glycero-&beta;-D-manno-heptose biosynthesis | 107.225139 | 406.1104235 | 71.37103258 | 442.8690419 | 31.8017254 | 379.944547 | 11.0566537 | 424.5859173 | 15.9008627 | 189.972274 | 5.52832686 | 212.2929587 | 0.043227 | * |
| aromatic biogenic amine degradation (bacteria) | 5.458779509 | 20.76682298 | 0 | 2.001530939 | 6.28220185 | 15.2885797 | 0 | 2.339617535 | 3.14110092 | 7.64428986 | 0 | 1.169808767 | 0.043227 | * |
| Bifidobacterium shunt | 1330.813315 | 299.6982455 | 1108.729355 | 170.1430354 | 495.33049 | 364.997604 | 540.029376 | 102.3590887 | 247.665245 | 182.498802 | 270.014688 | 51.17954435 | 0.043227 | * |
| biotin biosynthesis I | 601.3285669 | 1241.672094 | 533.088277 | 748.2874105 | 220.451957 | 664.861276 | 357.454246 | 111.7195242 | 110.225978 | 332.430638 | 178.727123 | 55.85976209 | 0.043227 | * |
| biotin biosynthesis II | 28.98589249 | 29.90487125 | 2.391183402 | 2.995113581 | 7.72642056 | 31.4072035 | 4.7823668 | 3.59353721 | 3.86321028 | 15.7036018 | 2.3911834 | 1.796768605 | 0.043895 | * |
| chorismate biosynthesis from 3-dehydroquinate | 1896.3756 | 3322.493333 | 1688.250658 | 2380.868929 | 559.868064 | 1131.80742 | 280.986801 | 215.9963082 | 279.934032 | 565.903711 | 140.4934 | 107.9981541 | 0.043227 | * |
| chorismate biosynthesis I | 1765.916155 | 2877.604187 | 1499.280548 | 2269.816462 | 583.584039 | 850.713618 | 324.866096 | 180.4113002 | 291.792019 | 425.356809 | 162.433048 | 90.20565011 | 0.045397 | * |
| coenzyme A biosynthesis I | 2262.548282 | 3190.958593 | 1977.9324 | 2458.636367 | 512.552851 | 1019.14611 | 358.940024 | 134.276824 | 256.276426 | 509.573056 | 179.470012 | 67.138412 | 0.047945 | * |
| colanic acid building blocks biosynthesis | 1289.923321 | 1713.834351 | 1218.027933 | 1640.639626 | 281.432169 | 246.559627 | 250.503197 | 84.06159125 | 140.716085 | 123.279814 | 125.251599 | 42.03079563 | 0.045397 | * |
| dTDP-L-rhamnose biosynthesis I | 1920.955938 | 3174.419981 | 1743.974161 | 2397.900072 | 546.914048 | 1014.6438 | 215.297794 | 222.2071182 | 273.457024 | 507.3219 | 107.648897 | 111.1035591 | 0.043227 | * |
| flavin biosynthesis I (bacteria and plants) | 1505.892471 | 2776.53106 | 1491.308712 | 2102.034912 | 379.772063 | 1032.21219 | 286.181882 | 243.9184158 | 189.886031 | 516.106095 | 143.090941 | 121.9592079 | 0.043227 | * |
| GDP-mannose biosynthesis | 1665.558888 | 3016.524004 | 1644.277207 | 2305.543333 | 440.734037 | 1250.84649 | 273.419469 | 147.3921731 | 220.367018 | 625.423247 | 136.709734 | 73.69608654 | 0.043227 | * |
| gluconeogenesis I | 2152.363252 | 2955.044811 | 1941.187454 | 2343.16688 | 478.521374 | 699.620613 | 317.478139 | 205.8568257 | 239.260687 | 349.810306 | 158.73907 | 102.9284129 | 0.045126 | * |
| glucose and glucose-1-phosphate degradation | 88.11358818 | 351.315108 | 26.83324147 | 834.4669786 | 34.566063 | 573.441845 | 33.6058303 | 499.9933505 | 17.2830315 | 286.720922 | 16.8029151 | 249.9966753 | 0.043227 | * |
| glycerol degradation to butanol | 422.2960918 | 116.9038654 | 251.8077252 | 47.80179111 | 247.868182 | 58.8567221 | 148.224815 | 55.253151 | 123.934091 | 29.4283611 | 74.1124077 | 27.6265755 | 0.043227 | * |
| glycogen degradation I (bacterial) | 1773.480494 | 2798.378658 | 1689.001145 | 2333.072037 | 487.488453 | 796.712702 | 299.490215 | 278.3241238 | 243.744226 | 398.356351 | 149.745107 | 139.1620619 | 0.043227 | * |
| heterolactic fermentation | 1049.307182 | 233.9493083 | 913.2180438 | 146.3655405 | 376.346102 | 270.618988 | 447.988017 | 86.47725999 | 188.173051 | 135.309494 | 223.994009 | 43.23863 | 0.043227 | * |
| hexitol fermentation to lactate, formate, ethanol and acetate | 326.3297655 | 161.8662036 | 196.1413093 | 43.30784393 | 77.2472837 | 72.7504836 | 97.3319976 | 37.74138918 | 38.6236418 | 36.3752418 | 48.6659988 | 18.87069459 | 0.043227 | * |
| incomplete reductive TCA cycle | 2243.267203 | 3518.630275 | 2142.309689 | 2805.256201 | 476.4685 | 1408.34492 | 282.722454 | 160.2587163 | 238.23425 | 704.172462 | 141.361227 | 80.12935814 | 0.047945 | * |
| L-arginine biosynthesis I (via L-ornithine) | 1695.335329 | 2327.558373 | 1348.295804 | 1958.305966 | 468.466421 | 578.353597 | 221.817204 | 70.09451366 | 234.23321 | 289.176798 | 110.908602 | 35.04725683 | 0.043227 | * |
| L-arginine biosynthesis II (acetyl cycle) | 1524.186531 | 2137.744108 | 1039.827731 | 1970.727452 | 510.290242 | 617.481328 | 203.962255 | 195.0810383 | 255.145121 | 308.740664 | 101.981128 | 97.54051915 | 0.043227 | * |
| L-arginine biosynthesis IV (archaebacteria) | 1703.075708 | 2333.392138 | 1353.001893 | 1959.02525 | 469.601401 | 581.242254 | 224.058562 | 67.94794514 | 234.8007 | 290.621127 | 112.029281 | 33.97397257 | 0.043227 | * |
| L-histidine biosynthesis | 1763.178722 | 2570.549941 | 1612.11379 | 2311.706312 | 552.130105 | 595.116927 | 266.929708 | 268.5299779 | 276.065052 | 297.558463 | 133.464854 | 134.2649889 | 0.048717 | * |
| L-isoleucine biosynthesis I (from threonine) | 2241.185975 | 3242.088286 | 2010.61501 | 2739.816059 | 660.223212 | 864.638007 | 282.318109 | 297.7939868 | 330.111606 | 432.319003 | 141.159054 | 148.8969934 | 0.043227 | * |
| L-isoleucine biosynthesis II | 2402.137968 | 3474.736974 | 2159.791313 | 2975.343197 | 710.192179 | 907.028236 | 308.642034 | 313.9977168 | 355.096089 | 453.514118 | 154.321017 | 156.9988584 | 0.043227 | * |
| L-isoleucine biosynthesis III | 2072.176577 | 2998.875376 | 1859.884064 | 2600.927074 | 604.914912 | 731.843192 | 300.973617 | 252.8550754 | 302.457456 | 365.921596 | 150.486808 | 126.4275377 | 0.043227 | * |
| L-isoleucine biosynthesis IV | 2310.918075 | 3360.439098 | 2044.686078 | 2783.613613 | 657.467283 | 885.284882 | 281.899807 | 256.6664928 | 328.733641 | 442.642441 | 140.949904 | 128.3332464 | 0.043895 | * |
| L-lysine biosynthesis II | 1274.186952 | 286.8356364 | 951.3442558 | 201.8132184 | 435.093773 | 312.687656 | 491.642869 | 90.37232863 | 217.546886 | 156.343828 | 245.821434 | 45.18616431 | 0.043227 | * |
| L-ornithine biosynthesis | 1285.852212 | 2043.464952 | 1229.566975 | 2035.239591 | 415.783983 | 453.390601 | 172.231484 | 138.4185168 | 207.891991 | 226.695301 | 86.1157421 | 69.20925842 | 0.043227 | * |
| L-tryptophan biosynthesis | 1379.084167 | 2261.663034 | 1451.659547 | 2245.796205 | 369.067701 | 673.846561 | 278.168035 | 131.7707623 | 184.533851 | 336.923281 | 139.084018 | 65.88538114 | 0.043227 | * |
| L-valine biosynthesis | 2241.185975 | 3242.088286 | 2010.61501 | 2739.816059 | 660.223212 | 864.638007 | 282.318109 | 297.7939868 | 330.111606 | 432.319003 | 141.159054 | 148.8969934 | 0.043227 | * |
| lactose and galactose degradation I | 509.9080287 | 37.41750439 | 342.1461173 | 26.11160704 | 240.835743 | 6.30053877 | 288.862582 | 33.20748947 | 120.417872 | 3.15026939 | 144.431291 | 16.60374474 | 0.043227 | * |
| methylerythritol phosphate pathway I | 1811.889879 | 2754.929405 | 1625.118566 | 2314.580061 | 554.828675 | 630.733035 | 265.267391 | 209.3246395 | 277.414338 | 315.366518 | 132.633695 | 104.6623198 | 0.043227 | * |
| methylerythritol phosphate pathway II | 1811.889879 | 2754.929405 | 1625.118566 | 2314.580061 | 554.828675 | 630.733035 | 265.267391 | 209.3246395 | 277.414338 | 315.366518 | 132.633695 | 104.6623198 | 0.043227 | * |
| N10-formyl-tetrahydrofolate biosynthesis | 1960.874797 | 3046.888205 | 1774.253337 | 2232.106268 | 449.058678 | 1020.1773 | 287.426417 | 145.2411271 | 224.529339 | 510.08865 | 143.713209 | 72.62056357 | 0.043784 | * |
| NAD biosynthesis I (from aspartate) | 1471.379348 | 2226.410443 | 1436.262612 | 2107.854727 | 410.884366 | 256.619949 | 271.75857 | 251.1549979 | 205.442183 | 128.309975 | 135.879285 | 125.577499 | 0.043227 | * |
| pantothenate and coenzyme A biosynthesis I | 1552.725588 | 2691.42759 | 1495.572989 | 2129.255959 | 324.276799 | 1027.40968 | 193.856835 | 73.85307464 | 162.138399 | 513.704842 | 96.9284173 | 36.92653732 | 0.043227 | * |
| peptidoglycan biosynthesis IV (Enterococcus faecium) | 1619.763422 | 915.9131958 | 1262.343905 | 553.5620612 | 381.187389 | 311.242263 | 392.309275 | 194.7439162 | 190.593695 | 155.621132 | 196.154638 | 97.3719581 | 0.043227 | * |
| peptidoglycan biosynthesis V (&beta;-lactam resistance) | 124.9054967 | 61.10477206 | 151.1335518 | 9.146800186 | 107.03847 | 44.2612706 | 13.8599567 | 18.29360037 | 53.5192349 | 22.1306353 | 6.92997833 | 9.146800186 | 0.043227 | * |
| photorespiration | 81.22727598 | 79.43494045 | 0 | 9.778720133 | 21.0612713 | 78.392558 | 0 | 11.78191802 | 10.5306356 | 39.196279 | 0 | 5.89095901 | 0.043227 | * |
| polyisoprenoid biosynthesis (E. coli) | 1697.291756 | 2753.752295 | 1604.115966 | 2131.984368 | 324.173242 | 1047.75013 | 221.832128 | 137.9166307 | 162.086621 | 523.875066 | 110.916064 | 68.95831536 | 0.043227 | * |
| polymyxin resistance | 0 | 16.1379463 | 3.386587951 | 16.10589848 | 0 | 7.88304204 | 4.00891969 | 16.3356779 | 0 | 3.94152102 | 2.00445984 | 8.167838952 | 0.043227 | * |
| pyrimidine deoxyribonucleotides de novo biosynthesis III | 765.0037664 | 625.4621358 | 440.5633619 | 443.8838619 | 195.597376 | 184.523218 | 142.326264 | 63.75349518 | 97.7986881 | 92.2616092 | 71.1631322 | 31.87674759 | 0.049639 | * |
| starch degradation V | 1698.251287 | 2787.125215 | 1775.051797 | 2606.42343 | 488.263916 | 599.922519 | 251.743219 | 397.7892765 | 244.131958 | 299.96126 | 125.87161 | 198.8946382 | 0.043227 | * |
| sulfate reduction I (assimilatory) | 220.1897156 | 1186.654402 | 145.4807499 | 916.65911 | 86.2774862 | 1231.6345 | 41.5497878 | 499.4581783 | 43.1387431 | 615.817249 | 20.7748939 | 249.7290891 | 0.043227 | * |
| superpathway of arginine and polyamine biosynthesis | 563.5404471 | 180.6745072 | 453.0659423 | 123.1680282 | 333.253342 | 83.2715215 | 187.614246 | 87.8313989 | 166.626671 | 41.6357608 | 93.8071228 | 43.91569945 | 0.043227 | * |
| superpathway of aromatic amino acid biosynthesis | 1833.530842 | 3030.367697 | 1581.02558 | 2420.857507 | 597.308025 | 866.361735 | 337.224175 | 182.1444394 | 298.654013 | 433.180867 | 168.612087 | 91.0722197 | 0.043227 | * |
| superpathway of branched amino acid biosynthesis | 2208.220717 | 3196.738597 | 1955.909287 | 2727.698553 | 628.389292 | 725.269643 | 312.993348 | 222.8308498 | 314.194646 | 362.634822 | 156.496674 | 111.4154249 | 0.043227 | * |
| superpathway of GDP-mannose-derived O-antigen building blocks biosynthesis | 1237.045104 | 2465.88642 | 1308.468731 | 1792.103538 | 268.438343 | 1110.82389 | 160.981429 | 224.3558126 | 134.219171 | 555.411945 | 80.4907146 | 112.1779063 | 0.043227 | * |
| superpathway of geranylgeranyl diphosphate biosynthesis II (via MEP) | 1893.864034 | 2802.084966 | 1692.138594 | 2310.052725 | 544.571011 | 632.474826 | 273.113262 | 211.2133703 | 272.285505 | 316.237413 | 136.556631 | 105.6066852 | 0.043227 | * |
| superpathway of glycolysis, pyruvate dehydrogenase, TCA, and glyoxylate bypass | 1.912465578 | 74.46540073 | 3.638485434 | 58.6809801 | 3.82493116 | 60.9501487 | 7.27697087 | 55.67369237 | 1.91246558 | 30.4750743 | 3.63848543 | 27.83684618 | 0.043227 | * |
| superpathway of glyoxylate bypass and TCA | 0.96016083 | 39.04600284 | 1.855876642 | 33.03538145 | 1.92032166 | 32.0299566 | 3.71175328 | 32.04451133 | 0.96016083 | 16.0149783 | 1.85587664 | 16.02225566 | 0.043227 | * |
| superpathway of L-phenylalanine biosynthesis | 36.1358897 | 158.17342 | 68.80871071 | 248.908287 | 19.3040482 | 17.4560294 | 62.8732507 | 148.5171034 | 9.65202408 | 8.72801469 | 31.4366254 | 74.2585517 | 0.043227 | * |
| superpathway of L-tyrosine biosynthesis | 36.09245711 | 157.5920312 | 68.26118809 | 246.6496179 | 19.2760951 | 17.5325873 | 62.2231265 | 147.007097 | 9.63804755 | 8.76629366 | 31.1115632 | 73.50354849 | 0.043227 | * |
| superpathway of phospholipid biosynthesis I (bacteria) | 2050.525695 | 2810.545613 | 1766.089904 | 2237.467053 | 469.097148 | 676.549577 | 291.021364 | 93.71281639 | 234.548574 | 338.274788 | 145.510682 | 46.85640819 | 0.043227 | * |
| superpathway of polyamine biosynthesis I | 348.8094606 | 95.20357611 | 278.0555327 | 64.50160927 | 223.250492 | 46.6269457 | 127.939947 | 46.26212384 | 111.625246 | 23.3134729 | 63.9699735 | 23.13106192 | 0.043227 | * |
| superpathway of polyamine biosynthesis II | 279.5819121 | 87.69253073 | 124.4902169 | 68.66413483 | 144.349273 | 49.2093383 | 47.0315201 | 49.29908465 | 72.1746364 | 24.6046691 | 23.51576 | 24.64954232 | 0.045769 | * |
| superpathway of sulfate assimilation and cysteine biosynthesis | 432.2505294 | 1359.949745 | 293.3317362 | 1233.928463 | 163.648922 | 1053.44339 | 74.9890535 | 527.5119995 | 81.8244608 | 526.721693 | 37.4945267 | 263.7559998 | 0.043227 | * |
| superpathway of UDP-glucose-derived O-antigen building blocks biosynthesis | 171.7806234 | 461.112136 | 164.7937355 | 576.3157553 | 83.1226395 | 86.2176796 | 59.6403904 | 292.7666144 | 41.5613198 | 43.1088398 | 29.8201952 | 146.3833072 | 0.043227 | * |
| taxadiene biosynthesis (engineered) | 1342.953351 | 1346.145997 | 1116.212635 | 518.4950012 | 356.559022 | 1119.03821 | 301.897374 | 101.0211884 | 178.279511 | 559.519105 | 150.948687 | 50.51059422 | 0.043227 | * |
| TCA cycle I (prokaryotic) | 744.4298567 | 1060.646358 | 403.7194363 | 1641.247086 | 280.858054 | 582.468742 | 218.67467 | 543.7540354 | 140.429027 | 291.234371 | 109.337335 | 271.8770177 | 0.043227 | * |
| TCA cycle IV (2-oxoglutarate decarboxylase) | 3.663668111 | 71.90450891 | 3.676266687 | 74.37953288 | 7.32733622 | 46.9254944 | 7.35253337 | 69.38431036 | 3.66366811 | 23.4627472 | 3.67626669 | 34.69215518 | 0.043227 | * |
| TCA cycle V (2-oxoglutarate:ferredoxin oxidoreductase) | 1212.946327 | 1731.57358 | 707.4178272 | 2033.417484 | 405.539873 | 379.999455 | 293.583197 | 318.6399837 | 202.769937 | 189.999728 | 146.791598 | 159.3199919 | 0.043227 | * |
| TCA cycle VI (obligate autotrophs) | 418.3054554 | 694.2764037 | 241.6616099 | 1439.666502 | 180.066185 | 680.119637 | 131.517145 | 680.5653483 | 90.0330923 | 340.059818 | 65.7585726 | 340.2826741 | 0.043784 | * |
| tetrapyrrole biosynthesis I (from glutamate) | 608.1865402 | 1035.654708 | 460.7119357 | 1134.612292 | 224.738569 | 440.94541 | 142.048416 | 258.3789833 | 112.369284 | 220.472705 | 71.0242079 | 129.1894916 | 0.043227 | * |
| tetrapyrrole biosynthesis II (from glycine) | 482.5202597 | 919.6598012 | 387.5508506 | 1028.920278 | 203.224351 | 483.268219 | 145.020721 | 253.6189617 | 101.612176 | 241.63411 | 72.5103606 | 126.8094809 | 0.043227 | * |
| UMP biosynthesis | 2521.239733 | 3510.783683 | 2218.811012 | 2721.599418 | 572.357049 | 1004.80479 | 392.209345 | 185.587433 | 286.178525 | 502.402396 | 196.104673 | 92.79371651 | 0.043895 | * |

**Table S8 Significantly changed predicted pathways at day 12**

| **Pathways** | **mean_Control** | **mean_Hydrogel** | **mean_*S.aureus*** | **mean_*S.aureus*+Hydrogel** | **sd_Control** | **sd_Hydrogel** | **sd_*S.aureus*** | **sd_*S.aureus*+Hydrogel** | **se_Control** | **se_Hydrogel** | **se_*S.aureus*** | **se_*S.aureus*+Hydrogel** | ***p* value adjusted** | **sign** |
| --- | --- | --- | --- | --- | --- | --- | --- | --- | --- | --- | --- | --- | --- | --- |
| chondroitin sulfate degradation I (bacterial) | 47.76819323 | 0 | 0 | 9.060005287 | 15.9401519 | 0 | 0 | 11.80576475 | 7.970075957 | 0 | 0 | 5.902882374 | 0.049639 | * |
| tRNA processing | 37.51551701 | 225.0872448 | 63.88584841 | 338.8562364 | 48.2663972 | 55.51872617 | 71.57156152 | 154.6082326 | 24.13319862 | 27.75936309 | 35.78578076 | 77.30411631 | 0.049639 | * |
| 1,4-dihydroxy-6-naphthoate biosynthesis I | 204.125 | 308.7483888 | 194.875 | 855.2554676 | 59.6341275 | 127.5729257 | 133.2393679 | 670.6875382 | 29.81706377 | 63.78646287 | 66.61968397 | 335.3437691 | 0.049639 | * |
| 1,4-dihydroxy-6-naphthoate biosynthesis II | 204.125 | 311.9175 | 194.875 | 856.4175 | 59.6341275 | 131.4916749 | 133.2393679 | 669.9667533 | 29.81706377 | 65.74583743 | 66.61968397 | 334.9833767 | 0.049639 | * |
| 5-aminoimidazole ribonucleotide biosynthesis I | 2715.658845 | 3914.569067 | 3245.993372 | 6116.094748 | 986.547473 | 987.0292035 | 1064.375845 | 2462.110356 | 493.2737364 | 493.5146018 | 532.1879224 | 1231.055178 | 0.049639 | * |
| 5-aminoimidazole ribonucleotide biosynthesis II | 2602.842287 | 3876.619378 | 3201.962742 | 6159.442389 | 957.735441 | 997.1888321 | 1078.605867 | 2632.368861 | 478.8677206 | 498.5944161 | 539.3029333 | 1316.184431 | 0.049639 | * |
| acetylene degradation | 903.3447086 | 2079.096234 | 2491.168608 | 4163.671606 | 714.328496 | 785.223736 | 978.9896217 | 3266.891553 | 357.1642482 | 392.611868 | 489.4948109 | 1633.445777 | 0.049639 | * |
| adenosine deoxyribonucleotides de novo biosynthesis II | 2240.865075 | 3381.447298 | 3484.364439 | 5974.57381 | 881.488615 | 915.1325725 | 1095.172815 | 3151.003016 | 440.7443075 | 457.5662862 | 547.5864075 | 1575.501508 | 0.049639 | * |
| adenosine ribonucleotides de novo biosynthesis | 2656.980572 | 4155.820403 | 3637.10088 | 6339.3393 | 962.76061 | 1171.217133 | 1268.468192 | 2615.004475 | 481.380305 | 585.6085663 | 634.2340961 | 1307.502238 | 0.049639 | * |
| adenosylcobalamin biosynthesis from cobyrinate a,c-diamide I | 524.1495079 | 1626.829813 | 907.1388211 | 2237.677565 | 367.803258 | 651.0456472 | 511.5672477 | 922.9010049 | 183.901629 | 325.5228236 | 255.7836239 | 461.4505025 | 0.049639 | * |
| adenosylcobalamin salvage from cobinamide I | 561.936051 | 1720.184579 | 955.8415857 | 2400.750364 | 385.248322 | 644.7400301 | 525.4123445 | 917.3491698 | 192.6241611 | 322.3700151 | 262.7061723 | 458.6745849 | 0.049639 | * |
| adenosylcobalamin salvage from cobinamide II | 526.7579752 | 1632.152075 | 911.0580666 | 2243.859461 | 368.457653 | 654.0178038 | 514.7588341 | 927.6206257 | 184.2288266 | 327.0089019 | 257.379417 | 463.8103128 | 0.049639 | * |
| ADP-L-glycero-&beta;-D-manno-heptose biosynthesis | 78.33725458 | 237.4656538 | 169.1499313 | 320.294807 | 51.559024 | 77.90281556 | 94.28114577 | 138.3248441 | 25.77951199 | 38.95140778 | 47.14057288 | 69.16242203 | 0.049639 | * |
| arginine, ornithine and proline interconversion | 138.1449173 | 457.1496448 | 224.0645011 | 518.8984459 | 97.3872549 | 112.5496062 | 163.0264923 | 69.36532128 | 48.69362743 | 56.27480309 | 81.51324615 | 34.68266064 | 0.049639 | * |
| aromatic biogenic amine degradation (bacteria) | 3.392746545 | 96.2216966 | 33.87503994 | 53.08598421 | 5.16399227 | 35.363565 | 53.54478308 | 28.11089403 | 2.581996134 | 17.6817825 | 26.77239154 | 14.05544702 | 0.049639 | * |
| biotin biosynthesis II | 9.536849573 | 51.25359174 | 40.9264169 | 135.8565852 | 11.6769763 | 41.08438544 | 42.00180698 | 59.32682313 | 5.838488156 | 20.54219272 | 21.00090349 | 29.66341157 | 0.049639 | * |
| Calvin-Benson-Bassham cycle | 2405.888136 | 4029.551132 | 3318.681805 | 5394.440673 | 915.38266 | 1289.161781 | 1383.630132 | 1785.052772 | 457.6913299 | 644.5808904 | 691.8150662 | 892.5263861 | 0.049639 | * |
| chorismate biosynthesis from 3-dehydroquinate | 2195.729609 | 3628.474703 | 2610.373595 | 4801.78131 | 766.558329 | 1028.153102 | 1003.75371 | 1339.913268 | 383.2791645 | 514.076551 | 501.8768552 | 669.9566338 | 0.049639 | * |
| chorismate biosynthesis I | 1820.977725 | 3559.62209 | 2485.528278 | 4759.113686 | 738.554932 | 1090.464404 | 1074.304571 | 1403.241184 | 369.2774662 | 545.2322021 | 537.1522857 | 701.6205922 | 0.049639 | * |
| cis-vaccenate biosynthesis | 2543.63423 | 3912.793804 | 3200.422389 | 5969.294641 | 905.779229 | 1024.615263 | 1071.721866 | 2310.106202 | 452.8896147 | 512.3076317 | 535.860933 | 1155.053101 | 0.049639 | * |
| fatty acid elongation -- saturated | 2340.906769 | 2662.145856 | 2565.110851 | 4569.933817 | 783.498309 | 319.9183109 | 624.7172811 | 1677.715394 | 391.7491547 | 159.9591554 | 312.3586406 | 838.8576969 | 0.049639 | * |
| flavin biosynthesis I (bacteria and plants) | 1911.100256 | 2301.471633 | 1920.621717 | 3626.172292 | 599.922797 | 468.1958109 | 522.8869595 | 924.422182 | 299.9613986 | 234.0979054 | 261.4434797 | 462.211091 | 0.049639 | * |
| galactose degradation I (Leloir pathway) | 1413.420183 | 2712.670929 | 2705.145898 | 4489.897788 | 788.655394 | 885.1734433 | 1006.707852 | 2301.240319 | 394.3276972 | 442.5867216 | 503.353926 | 1150.62016 | 0.049639 | * |
| glycogen biosynthesis I (from ADP-D-Glucose) | 1748.466877 | 3677.510911 | 2862.902895 | 5576.480544 | 938.825033 | 1173.152357 | 1196.570665 | 2602.567695 | 469.4125167 | 586.5761783 | 598.2853324 | 1301.283847 | 0.049639 | * |
| glycogen degradation I (bacterial) | 2125.796346 | 3561.074436 | 2689.166144 | 4858.935368 | 849.287749 | 1189.439711 | 1075.481352 | 1806.886705 | 424.6438746 | 594.7198554 | 537.7406761 | 903.4433525 | 0.049639 | * |
| gondoate biosynthesis (anaerobic) | 2617.52316 | 4025.703991 | 3368.638604 | 6217.422048 | 933.319877 | 1055.496025 | 1076.957628 | 2505.441849 | 466.6599386 | 527.7480124 | 538.4788142 | 1252.720925 | 0.049639 | * |
| guanosine deoxyribonucleotides de novo biosynthesis II | 2240.865075 | 3381.447298 | 3484.364439 | 5974.57381 | 881.488615 | 915.1325725 | 1095.172815 | 3151.003016 | 440.7443075 | 457.5662862 | 547.5864075 | 1575.501508 | 0.049639 | * |
| heme biosynthesis I (aerobic) | 70.33545541 | 122.0845998 | 44.9109111 | 247.2260834 | 66.8082121 | 37.64658172 | 41.55171156 | 133.4945479 | 33.40410606 | 18.82329086 | 20.77585578 | 66.74727394 | 0.049639 | * |
| inosine-5'-phosphate biosynthesis I | 2289.680665 | 3458.607032 | 2860.470762 | 5375.980594 | 819.056373 | 925.5891441 | 959.8379702 | 2178.948497 | 409.5281867 | 462.7945721 | 479.9189851 | 1089.474248 | 0.049639 | * |
| L-arginine biosynthesis I (via L-ornithine) | 1580.485944 | 3092.772136 | 2220.979097 | 4515.374857 | 632.752059 | 949.5888994 | 887.5235807 | 1735.843849 | 316.3760295 | 474.7944497 | 443.7617903 | 867.9219244 | 0.049639 | * |
| L-arginine biosynthesis II (acetyl cycle) | 1251.409368 | 3035.893063 | 2042.050771 | 4326.076977 | 692.418475 | 976.3936932 | 992.9290405 | 1671.803631 | 346.2092377 | 488.1968466 | 496.4645202 | 835.9018154 | 0.049639 | * |
| L-arginine biosynthesis III (via N-acetyl-L-citrulline) | 1254.561478 | 1851.347474 | 1409.871241 | 2541.934129 | 524.821004 | 333.2231257 | 451.9704877 | 698.7472688 | 262.410502 | 166.6115628 | 225.9852439 | 349.3736344 | 0.049639 | * |
| L-arginine biosynthesis IV (archaebacteria) | 1588.052348 | 3095.516125 | 2226.369101 | 4520.436939 | 637.488338 | 945.7976922 | 887.5780529 | 1726.063406 | 318.7441691 | 472.8988461 | 443.7890265 | 863.0317031 | 0.049639 | * |
| L-histidine biosynthesis | 2064.428601 | 3246.020467 | 2260.860163 | 4375.780991 | 707.779837 | 880.0985523 | 830.5276721 | 1095.971789 | 353.8899185 | 440.0492761 | 415.263836 | 547.9858947 | 0.049639 | * |
| L-isoleucine biosynthesis I (from threonine) | 2556.877187 | 4352.433357 | 3045.691378 | 5996.757507 | 949.977892 | 1292.069414 | 1279.638256 | 2037.7227 | 474.9889462 | 646.0347071 | 639.8191282 | 1018.86135 | 0.049639 | * |
| L-isoleucine biosynthesis II | 2750.837209 | 4680.958723 | 3263.957721 | 6384.607092 | 1013.75471 | 1402.456697 | 1368.001596 | 2085.297497 | 506.8773568 | 701.2283486 | 684.0007981 | 1042.648748 | 0.049639 | * |
| L-isoleucine biosynthesis III | 2384.106313 | 3943.486276 | 2766.43318 | 5368.33879 | 850.969387 | 1162.271214 | 1114.685026 | 1628.249003 | 425.4846934 | 581.1356072 | 557.3425129 | 814.1245014 | 0.049639 | * |
| L-isoleucine biosynthesis IV | 2580.781589 | 4474.482543 | 3157.852603 | 6086.76234 | 944.02142 | 1325.085606 | 1366.990009 | 1966.9366 | 472.0107098 | 662.542803 | 683.4950045 | 983.4682999 | 0.049639 | * |
| L-lysine biosynthesis I | 1196.519555 | 3036.729302 | 2252.058445 | 4365.866406 | 665.701903 | 1114.692117 | 1033.653727 | 1941.714865 | 332.8509513 | 557.3460585 | 516.8268637 | 970.8574324 | 0.049639 | * |
| L-lysine biosynthesis III | 2486.714775 | 3953.969027 | 3221.072007 | 6010.024208 | 926.389302 | 1109.721569 | 1124.330769 | 2442.979265 | 463.1946511 | 554.8607845 | 562.1653845 | 1221.489632 | 0.049639 | * |
| L-lysine biosynthesis VI | 2446.725883 | 3899.995411 | 3190.527365 | 5920.070256 | 907.08356 | 1101.008455 | 1120.715854 | 2471.900564 | 453.5417798 | 550.5042273 | 560.3579272 | 1235.950282 | 0.049639 | * |
| L-methionine biosynthesis III | 544.0902079 | 822.1326413 | 755.1575265 | 1839.768105 | 80.8839198 | 256.2373309 | 389.1720397 | 1183.859669 | 40.44195988 | 128.1186654 | 194.5860198 | 591.9298345 | 0.049639 | * |
| L-ornithine biosynthesis | 1637.398834 | 2680.622507 | 1765.950317 | 3538.317168 | 529.544165 | 872.8650757 | 736.9936434 | 1126.219249 | 264.7720824 | 436.4325379 | 368.4968217 | 563.1096246 | 0.049639 | * |
| L-valine biosynthesis | 2556.877187 | 4352.433357 | 3045.691378 | 5996.757507 | 949.977892 | 1292.069414 | 1279.638256 | 2037.7227 | 474.9889462 | 646.0347071 | 639.8191282 | 1018.86135 | 0.049639 | * |
| methylerythritol phosphate pathway I | 2128.377076 | 3406.844198 | 2519.649417 | 4671.831218 | 751.034454 | 1020.060048 | 983.8571308 | 1345.536609 | 375.5172268 | 510.0300238 | 491.9285654 | 672.7683046 | 0.049639 | * |
| methylerythritol phosphate pathway II | 2128.377076 | 3406.844198 | 2519.649417 | 4671.831218 | 751.034454 | 1020.060048 | 983.8571308 | 1345.536609 | 375.5172268 | 510.0300238 | 491.9285654 | 672.7683046 | 0.049639 | * |
| mevalonate pathway I | 224.0975221 | 188.2058097 | 806.3878627 | 937.2196237 | 152.009131 | 107.4122054 | 266.5388017 | 1376.503847 | 76.00456532 | 53.70610269 | 133.2694009 | 688.2519234 | 0.049639 | * |
| N10-formyl-tetrahydrofolate biosynthesis | 2128.930334 | 3182.839128 | 2695.814614 | 4520.778393 | 750.934695 | 820.2597104 | 906.3887681 | 1661.665143 | 375.4673473 | 410.1298552 | 453.1943841 | 830.8325714 | 0.049639 | * |
| NAD biosynthesis I (from aspartate) | 1846.050721 | 2393.428264 | 1858.131517 | 3644.294098 | 586.938547 | 437.7273995 | 495.0361567 | 937.7320713 | 293.4692733 | 218.8636997 | 247.5180784 | 468.8660357 | 0.049639 | * |
| O-antigen building blocks biosynthesis (E. coli) | 1137.792661 | 2476.662409 | 2222.841077 | 3809.90569 | 571.864773 | 693.9045975 | 825.341095 | 1704.031875 | 285.9323865 | 346.9522987 | 412.6705475 | 852.0159373 | 0.049639 | * |
| pentose phosphate pathway (non-oxidative branch) | 2751.040681 | 5116.390888 | 4140.968294 | 7201.413226 | 1027.19279 | 1876.293179 | 1793.87718 | 2827.119985 | 513.5963954 | 938.1465896 | 896.93859 | 1413.559993 | 0.049639 | * |
| peptidoglycan biosynthesis I (meso-diaminopimelate containing) | 2360.19982 | 3648.704211 | 3252.262887 | 5698.63592 | 861.999202 | 961.5921051 | 1107.329249 | 2421.62384 | 430.9996009 | 480.7960525 | 553.6646245 | 1210.81192 | 0.049639 | * |
| peptidoglycan biosynthesis III (mycobacteria) | 2340.134884 | 3614.213571 | 3231.02063 | 5680.055274 | 864.342053 | 941.2242454 | 1106.128995 | 2424.859008 | 432.1710267 | 470.6121227 | 553.0644975 | 1212.429504 | 0.049639 | * |
| peptidoglycan biosynthesis V (&beta;-lactam resistance) | 0 | 265.4010387 | 140.1375871 | 542.0089014 | 0 | 468.8466404 | 61.51550591 | 550.1730166 | 0 | 234.4233202 | 30.75775295 | 275.0865083 | 0.049639 | * |
| phosphatidylglycerol biosynthesis I (plastidic) | 1374.503823 | 3186.232887 | 2791.060368 | 5028.702742 | 777.220376 | 1109.541978 | 1187.009789 | 2670.261969 | 388.6101882 | 554.7709892 | 593.5048946 | 1335.130984 | 0.049639 | * |
| phosphatidylglycerol biosynthesis II (non-plastidic) | 1374.503823 | 3186.232887 | 2791.060368 | 5028.702742 | 777.220376 | 1109.541978 | 1187.009789 | 2670.261969 | 388.6101882 | 554.7709892 | 593.5048946 | 1335.130984 | 0.049639 | * |
| photorespiration | 26.94716664 | 143.890054 | 115.3731605 | 348.4938827 | 33.8952198 | 108.8214319 | 116.4978076 | 143.9209354 | 16.94760989 | 54.41071595 | 58.24890379 | 71.96046772 | 0.049639 | * |
| purine nucleobases degradation I (anaerobic) | 266.10099 | 1349.397703 | 662.4999675 | 1301.508157 | 287.982103 | 949.9634155 | 444.9277556 | 569.7967475 | 143.9910515 | 474.9817078 | 222.4638778 | 284.8983737 | 0.049639 | * |
| pyrimidine deoxyribonucleotides de novo biosynthesis III | 501.5660847 | 859.7942831 | 767.897796 | 1549.358914 | 172.227645 | 234.2891916 | 403.1857316 | 693.1503057 | 86.11382231 | 117.1445958 | 201.5928658 | 346.5751529 | 0.049639 | * |
| pyruvate fermentation to acetate and lactate II | 2488.242345 | 4025.204296 | 3668.843142 | 6183.730554 | 950.641704 | 1117.916438 | 1388.784895 | 2780.348126 | 475.3208521 | 558.9582192 | 694.3924473 | 1390.174063 | 0.049639 | * |
| pyruvate fermentation to acetone | 266.5591445 | 1401.812183 | 430.0514708 | 1160.667749 | 124.872451 | 506.2076047 | 168.8902541 | 711.0792051 | 62.43622527 | 253.1038024 | 84.44512705 | 355.5396026 | 0.049639 | * |
| pyruvate fermentation to isobutanol (engineered) | 2521.671887 | 4558.492909 | 3362.022187 | 6296.408844 | 1014.9992 | 1555.224425 | 1450.526569 | 2326.842738 | 507.4995991 | 777.6122124 | 725.2632847 | 1163.421369 | 0.049639 | * |
| sucrose degradation IV (sucrose phosphorylase) | 37.71078411 | 426.7558312 | 538.9088321 | 755.4401622 | 27.8631353 | 297.7188357 | 502.2191595 | 819.4366747 | 13.93156764 | 148.8594178 | 251.1095797 | 409.7183373 | 0.049639 | * |
| superpathay of heme biosynthesis from glutamate | 131.4422589 | 267.5602009 | 103.1205292 | 512.4581085 | 113.038031 | 73.69237376 | 94.48848687 | 248.5580342 | 56.51901544 | 36.84618688 | 47.24424343 | 124.2790171 | 0.049639 | * |
| superpathway of 5-aminoimidazole ribonucleotide biosynthesis | 2602.842287 | 3876.619378 | 3201.962742 | 6159.442389 | 957.735441 | 997.1888321 | 1078.605867 | 2632.368861 | 478.8677206 | 498.5944161 | 539.3029333 | 1316.184431 | 0.049639 | * |
| superpathway of adenosine nucleotides de novo biosynthesis I | 2519.791063 | 3937.063636 | 3618.117087 | 6258.619955 | 949.49418 | 1102.224394 | 1238.213863 | 2801.174422 | 474.7470899 | 551.1121969 | 619.1069314 | 1400.587211 | 0.049639 | * |
| superpathway of aromatic amino acid biosynthesis | 1928.868716 | 3685.46281 | 2583.29522 | 4930.198078 | 767.354807 | 1145.844663 | 1103.30859 | 1439.24822 | 383.6774037 | 572.9223313 | 551.6542949 | 719.6241102 | 0.049639 | * |
| superpathway of branched amino acid biosynthesis | 2469.060983 | 4098.890986 | 2902.72816 | 5668.536581 | 885.703507 | 1188.838966 | 1162.660241 | 1798.563775 | 442.8517534 | 594.4194832 | 581.3301205 | 899.2818875 | 0.049639 | * |
| superpathway of geranylgeranyl diphosphate biosynthesis II (via MEP) | 2151.002405 | 3390.231916 | 2612.781587 | 4783.255399 | 761.374232 | 972.9101601 | 983.826492 | 1457.411578 | 380.6871159 | 486.45508 | 491.913246 | 728.7057889 | 0.049639 | * |
| superpathway of geranylgeranyldiphosphate biosynthesis I (via mevalonate) | 305.3973224 | 260.5517744 | 1031.464597 | 1185.746666 | 204.525287 | 146.0307727 | 336.8855548 | 1680.788418 | 102.2626435 | 73.01538635 | 168.4427774 | 840.3942088 | 0.049639 | * |
| superpathway of glycol metabolism and degradation | 0.630024253 | 52.2827389 | 7.647300278 | 80.15865539 | 1.26004851 | 14.25116494 | 10.90691783 | 74.97855987 | 0.630024253 | 7.125582469 | 5.453458917 | 37.48927993 | 0.049639 | * |
| superpathway of glycolysis and Entner-Doudoroff | 448.5895888 | 1695.824874 | 1155.396211 | 2207.751903 | 461.436255 | 477.1419067 | 686.1004631 | 925.344405 | 230.7181276 | 238.5709533 | 343.0502315 | 462.6722025 | 0.049639 | * |
| superpathway of hexitol degradation (bacteria) | 0 | 527.3473198 | 314.3130989 | 571.997963 | 0 | 344.5861749 | 256.8471131 | 375.5618439 | 0 | 172.2930875 | 128.4235566 | 187.780922 | 0.049639 | * |
| superpathway of L-alanine biosynthesis | 80.98655624 | 465.4265798 | 222.2207272 | 570.3703606 | 80.3185382 | 241.3213773 | 211.7525513 | 200.7309835 | 40.15926912 | 120.6606887 | 105.8762756 | 100.3654917 | 0.049639 | * |
| superpathway of L-isoleucine biosynthesis I | 2460.97358 | 3891.165071 | 2887.840487 | 5489.855222 | 896.084886 | 1107.770725 | 1073.472898 | 1819.943508 | 448.0424429 | 553.8853625 | 536.7364492 | 909.9717542 | 0.049639 | * |
| superpathway of L-lysine, L-threonine and L-methionine biosynthesis I | 615.3956059 | 1523.433361 | 1085.476104 | 1973.882028 | 382.500774 | 617.3479851 | 406.778707 | 765.0097303 | 191.250387 | 308.6739926 | 203.3893535 | 382.5048652 | 0.049639 | * |
| superpathway of L-methionine biosynthesis (by sulfhydrylation) | 213.1616446 | 513.8035635 | 241.2103994 | 712.6524467 | 108.970337 | 154.1995673 | 115.3699188 | 242.1951234 | 54.48516858 | 77.09978366 | 57.68495938 | 121.0975617 | 0.049639 | * |
| superpathway of L-phenylalanine biosynthesis | 8.241062069 | 508.5266256 | 150.2127642 | 377.4920908 | 9.87983227 | 523.5722378 | 165.2398065 | 502.6053858 | 4.939916136 | 261.7861189 | 82.61990323 | 251.3026929 | 0.049639 | * |
| superpathway of L-serine and glycine biosynthesis I | 2299.804041 | 3583.770355 | 2847.686415 | 5387.62342 | 800.900571 | 1068.65326 | 1060.76857 | 2115.697887 | 400.4502857 | 534.3266301 | 530.384285 | 1057.848944 | 0.049639 | * |
| superpathway of L-threonine biosynthesis | 2449.941034 | 3729.779117 | 2938.572132 | 5365.95687 | 896.402668 | 1040.96301 | 1024.402854 | 1847.747552 | 448.2013342 | 520.4815049 | 512.2014268 | 923.8737758 | 0.049639 | * |
| superpathway of L-tyrosine biosynthesis | 8.231519387 | 505.8145682 | 149.7970631 | 376.9725831 | 9.86837349 | 520.1232787 | 164.6881703 | 502.1032381 | 4.934186747 | 260.0616394 | 82.34408514 | 251.051619 | 0.049639 | * |
| superpathway of menaquinol-8 biosynthesis II | 482.7544749 | 673.4237879 | 458.8482616 | 1387.228493 | 125.068897 | 188.0156287 | 289.0970413 | 764.7801633 | 62.53444841 | 94.00781436 | 144.5485207 | 382.3900816 | 0.049639 | * |
| superpathway of N-acetylneuraminate degradation | 651.3922357 | 2056.111593 | 1612.440577 | 3040.929061 | 627.044429 | 595.4300237 | 901.9455702 | 1633.130113 | 313.5222143 | 297.7150118 | 450.9727851 | 816.5650565 | 0.049639 | * |
| superpathway of phospholipid biosynthesis I (bacteria) | 2025.768601 | 3446.129975 | 3086.983751 | 5449.79481 | 712.561897 | 1131.976705 | 1130.181336 | 2663.33204 | 356.2809486 | 565.9883524 | 565.0906682 | 1331.66602 | 0.049639 | * |
| superpathway of sulfur oxidation (Acidianus ambivalens) | 64.5 | 320.84 | 108 | 383.92 | 61.021854 | 175.1977382 | 112.4767105 | 154.8949717 | 30.510927 | 87.59886909 | 56.23835524 | 77.44748586 | 0.049639 | * |
| superpathway of taurine degradation | 0 | 0 | 0 | 20.45305174 | 0 | 0 | 0 | 32.07433575 | 0 | 0 | 0 | 16.03716787 | 0.049639 | * |
| superpathway of thiamin diphosphate biosynthesis I | 1923.324544 | 2056.188133 | 1678.691952 | 3115.262989 | 614.692733 | 262.3608606 | 335.7392591 | 942.9403861 | 307.3463667 | 131.1804303 | 167.8696296 | 471.470193 | 0.049639 | * |
| superpathway of thiamin diphosphate biosynthesis II | 423.1518306 | 1330.959078 | 813.3734013 | 1795.918833 | 353.005594 | 150.8539428 | 501.6916639 | 746.3279786 | 176.5027969 | 75.42697138 | 250.8458319 | 373.1639893 | 0.049639 | * |
| TCA cycle I (prokaryotic) | 815.1297814 | 1208.965791 | 777.34672 | 1659.365127 | 292.717962 | 317.1923339 | 361.646847 | 352.3311636 | 146.3589811 | 158.596167 | 180.8234235 | 176.1655818 | 0.049639 | * |
| TCA cycle V (2-oxoglutarate:ferredoxin oxidoreductase) | 1292.759249 | 1960.495021 | 1359.797199 | 2644.390164 | 432.414001 | 405.9667206 | 543.369989 | 456.6593319 | 216.2070003 | 202.9833603 | 271.6849945 | 228.3296659 | 0.049639 | * |
| TCA cycle VIII (helicobacter) | 173.1941357 | 304.680881 | 110.4848402 | 681.7194742 | 218.254885 | 185.0210351 | 95.66924678 | 285.419698 | 109.1274425 | 92.51051755 | 47.83462339 | 142.709849 | 0.049639 | * |
| tetrapyrrole biosynthesis I (from glutamate) | 600.8803965 | 1634.560815 | 1029.915461 | 2115.448622 | 363.048025 | 633.139453 | 623.6283831 | 674.4570487 | 181.5240124 | 316.5697265 | 311.8141915 | 337.2285244 | 0.049639 | * |
| tetrapyrrole biosynthesis II (from glycine) | 525.2205091 | 1463.593126 | 936.549689 | 1882.797803 | 365.054486 | 602.3724595 | 588.9156098 | 630.5096723 | 182.5272429 | 301.1862298 | 294.4578049 | 315.2548361 | 0.049639 | * |
| thiazole biosynthesis I (E. coli) | 1623.114793 | 2087.799193 | 1679.719299 | 2748.022955 | 596.492747 | 327.4799363 | 468.4780296 | 373.5498615 | 298.2463735 | 163.7399682 | 234.2390148 | 186.7749307 | 0.049639 | * |
| thiazole biosynthesis II (Bacillus) | 113.321205 | 560.6866563 | 303.2675138 | 651.8213789 | 109.156061 | 93.81889721 | 219.563008 | 292.1864627 | 54.57803053 | 46.9094486 | 109.781504 | 146.0932313 | 0.049639 | * |
| tRNA charging | 2352.149725 | 3623.060497 | 3139.371419 | 5481.889925 | 847.015992 | 981.2235496 | 1085.021366 | 2153.649998 | 423.5079958 | 490.6117748 | 542.510683 | 1076.824999 | 0.049639 | * |
| UDP-N-acetyl-D-glucosamine biosynthesis I | 993.3047954 | 2476.117832 | 2239.781095 | 3974.355445 | 571.75907 | 844.1704001 | 918.34955 | 2245.057482 | 285.8795348 | 422.0852001 | 459.174775 | 1122.528741 | 0.049639 | * |
| UDP-N-acetylmuramoyl-pentapeptide biosynthesis I (meso-diaminopimelate containing) | 2386.62497 | 3708.338803 | 3308.463695 | 5793.077592 | 878.805516 | 975.9723716 | 1132.215187 | 2500.689021 | 439.4027582 | 487.9861858 | 566.1075933 | 1250.344511 | 0.049639 | * |
| UDP-N-acetylmuramoyl-pentapeptide biosynthesis II (lysine-containing) | 2358.40308 | 3676.38021 | 3250.466555 | 5754.556074 | 872.502927 | 965.0994625 | 1126.487176 | 2422.031099 | 436.2514636 | 482.5497313 | 563.243588 | 1211.01555 | 0.049639 | * |
| UMP biosynthesis | 2557.694645 | 3943.238795 | 3504.54567 | 6042.00205 | 926.910838 | 1119.75255 | 1219.441446 | 2451.409108 | 463.4554189 | 559.876275 | 609.7207228 | 1225.704554 | 0.049639 | * |
